# An exploration of perinatal healthcare providers’ perspectives on respectful maternity care in the United States: a scoping review

**DOI:** 10.1186/s12884-025-08247-y

**Published:** 2025-11-04

**Authors:** Celestine Yayra Ofori-Parku, Orlando Omar Harris, Kimberly Baltzell, Emily Little, Ifeyinwa V. Asiodu

**Affiliations:** 1 Department of Family Health Care Nursing, School of Nursing, University of California San Francisco, 490 Illinois Street, #92J, San Francisco, CA 94158 USA; 2 Community Health Systems, School of Nursing, University of California San Francisco, 490 Illinois Street, #92S, San Francisco, CA 94158 USA; 3 Department of Family Health Care Nursing, School of Nursing, University of California San Francisco, 550 16th Street, #3507, San Francisco, CA, Oregon CA 94158 USA; 4 Nurturely, 56 East 15th Ave, Eugene, OR 97401 USA

**Keywords:** Respectful maternity care, Perinatal health providers, Mistreatment, Black women, Perinatal outcomes, Morbidity, Maternal mortality, Perinatal care, United states, Scoping review

## Abstract

**Background:**

Maternal mortality and morbidity rates in the United States (US) among racially minoritized populations have continued to worsen over the past decade. Reviews have examined the perinatal healthcare experiences and outcomes of Black individuals in the US. However, few reviews have examined perinatal healthcare providers’ experiences practicing in the US healthcare system. The purpose of this review was to comprehensively assess the current evidence and knowledge gaps related to perinatal healthcare providers’ perspectives on providing respectful maternity care to Black patients.

**Method:**

A literature search was conducted via PubMed, Embase, Web of Science, and CINAHL using appropriate search terms. This scoping review was conducted in accordance with the Preferred Reporting Items for Systematic Review and Meta-analysis extension for Scoping Reviews guidelines and by the Joanna Briggs Institute enhanced scoping review framework.

**Results:**

The first and second database searches yielded 764 and 819 articles respectively (2013–2023). An updated search yielded a total of 1,592 articles (2013–2024). Thirty-nine studies met full text review, 14 studies were ultimately included in this review (11 qualitative, two quantitative, and one mix-method). Thematic synthesis of studies in this review yielded a six-component typology of providers’ views and experiences on respectful maternity care in the US. The themes were (1) being free from harm and mistreatment such as physical and verbal abuse; (2) developing rapport between providers and women; (3) meeting professional standards of care such as seeking consent and not performing procedures against patients’ wishes; (4) avoiding discrimination based on age, race/ethnicity, and medical conditions, and low socioeconomic status; (5) health system constraints and facilitators; and (6) macro-level, external constraints, and facilitators.

**Conclusion:**

Findings from this review showed that providers’ descriptive narratives mirrors the body of evidence on individual pregnant women’s accounts of mistreatment while navigating perinatal care. However, none of the fourteen studies focused on providers admitting to their own practices that are or could be deemed disrespectful. Future research on the topic of this scoping review would benefit from looking at perinatal care providers’ willingness to admit to and be accountable for their disrespectful practices. More research is necessary to fully understand and disrupt dehumanizing perinatal care practices.

**Registration:**

This review has been registered with the Open Science Framework (10.17605/OSF.IO/DQXG2).

**Supplementary Information:**

The online version contains supplementary material available at 10.1186/s12884-025-08247-y.

## Introduction

Maternal mortality is a critical global health issue [1]. Despite the United States (US) spending the highest per capita on healthcare, comparative data shows that the US has the highest maternal mortality rate (32.9 per 100,000 live births) compared to other well- resourced countries in the global north [2–4]. For example, the Netherlands (1.2 per 100,000 live births), Australia (2.0 per 100,000 live births), Japan (2.7 per 100,000 live births), Germany (3.6 per 100,000 live births), Norway (3.7 per 100,000 live births), and the United Kingdom (6.5 per 100,000 live births) all have lower maternal mortality rates compared to the US [2–4]. Recent literature and reports in the US show that 817 women died from pregnancy or birthing complications in 2022 compared to 1,205 deaths in 2021, 861 in 2020, 754 in 2019, and 658 in 2018 [2, 5]. According to the CDC, in 2022, the highest maternal mortality rate in the US was in the southern states, with Mississippi leading at 82.5 deaths per 100,000 live births, while states like California and Massachusetts recorded lower rates, at 9.7 and 17.4, respectively [5]. However, when these statistics are analyzed by race and ethnicity, a stark disparity emerges, with Black women experiencing significantly higher maternal mortality rates compared to other racial groups [2, 5, 6]. Maternal mortality rates in the US among racially minoritized populations have continued to worsen over the past decade [7].

Maternal mortality rates for Black women have worsened more than any other racial groups between 2019 and 2021 [8, 9]. Maternal mortality rates among non-Hispanic Black or African American women was 44 per 100,000 live births in 2019 [2, 6]. These rates steadily increased to 55.3 per 100,000 live births in 2020 and 69.9 per 100,000 live births in 2021 [2, 6]. Although the US maternal mortality rate in 2022 decreased to 22.3 deaths per 100,000 live births, compared with a rate of 32.9 per 100,000 live births in 2021, the maternal mortality rate for Black women was still significantly higher: 49.5 deaths per 100,000 live births than rates for White (19.0), Hispanic (16.9), and Asian (13.2) women [5].

While the most tragic outcome for pregnant women or other pregnant people is death, severe maternal morbidity (SMM) can occur and affects roughly 55,000 women annually in the United States. SMM is 100 times more common than deaths [10, 11]. The SMM terminology refers to near misses or unforeseen adverse outcomes of labor and delivery such as acute myocardial infarction, blood product transfusions, aneurysm, acute renal failure, adult respiratory distress syndrome, hysterectomy, and amniotic fluid/air embolism that have immediate severe or long-term effects for the health of a birthing mother [12, 13]. A near miss is characterized as pregnant people who are critically ill or recently postpartum individuals who survived a life-threatening complication during pregnancy, childbirth, or within 42 days following the end of pregnancy [14]. Similar to the disparate rates of maternal mortality in the US, racial disparities exist in SMM during delivery, after discharge, and extending to postpartum re-hospitalizations [8]. At the state level, significant racial inequalities exist even in states like California, which has the lowest maternal mortality rate in the US. In California, the pregnancy-related mortality rate for Black women (45.8 deaths per 100,000) is about three to four times higher than that of other racial groups [15]. These issues are compounded by other intersectional disparities, such as racism, bias, discrimination, disrespectful care, and inequitable access to quality care [15].

Skilled maternity care through hospitals, clinics, or birthing centers— and in the case of low-risk pregnancies, planned home birth— throughout pregnancy, labor, and the immediate postpartum period is critical for addressing maternal mortality and morbidity [16, 17]. However, in the US where more than 98% of births occur in hospitals [18, 19], Black women tend to receive lower-quality maternity care and have poor health outcomes [16, 20–23]. Although pregnant people have a fundamental right to respectful maternity care, the opposite, disrespect, abuse, and mistreatment, is prevalent in many facility-based maternity care settings, creating adverse maternal outcomes especially for Black women [24–27]. Also, providers’ intentional or unintentional mistreatment [28, 29] of pregnant people during the perinatal period constitutes a violation of their human rights to respectful maternity care, which signifies poor-quality care [30].

Recent reviews have examined the perinatal healthcare experiences and outcomes of Black women in the US [31–33]. However, experience of care is only one—albeit important— aspect of quality maternity care [34, 35]. The other component pertains to the quality of care rendered by providers and healthcare systems.

Existing reviews on providers’ perspectives on perinatal care often focus on low and middle-income countries where facility-based maternity care utilization is lower than in the United States [36, 37]. Few reviews have examined providers’ experiences in the US, where more than 98% of births take place in hospitals [18, 19]. Providers’ interactions with pregnant and birthing people significantly impact how birthing people perceive and experience maternal health care [38]. Providing a comprehensive summary of providers’ perspectives on delivering facility-based maternity care that is free of discrimination and maltreatment can significantly inform policies and strategies to enhance quality and equitable maternity care services. Hence, a scoping review is an appropriate approach to understanding these experiences as it can help identify available evidence and determine whether further studies into the Black maternity care challenge are warranted [38]. The objective of this scoping review was to assess the current literature for scoping review and gaps in the literature related to perinatal healthcare providers’ perspectives on providing respectful maternity care to Black patients.

### Notes on terminology

Although the words “pregnant woman,” “postpartum women,” and related pronouns are used herein, we recognizes the existence of diverse gender identities and acknowledge that not all individuals who present for perinatal care self-identify as women or mothers. But for the purposes of this review, where we are referencing the published results of previous studies, terms used by the original authors are retained for accuracy.

### Description of authors

All authors are trained and/or experienced researchers. With our diverse backgrounds and shared commitment to equity in healthcare, we collaborated to create this manuscript. Author 1 identifies as a Black woman, a Nurse-Midwife, and a doctoral candidate. She is an experienced maternal health care equity advocate and perinatal health equity researcher. Author 2 is an Associate Professor of Nursing. He is an experienced sexual and gender minority researcher. He is also experienced in global public health, community-based participatory research, and working with socioeconomically marginalized groups. Author 3 identifies as a white woman and a Professor in the School of Nursing. She is an experienced quantitative and mixed methods researcher. She is also experienced in global health education, global health nursing education, quality of care in global health, and midwifery. Author 4 is a perinatal equity researcher, antiracism and climate action advocate, culture inclusion scientist and strategist. She also identifies as a white woman. Author 5 identifies as a Black woman and is an Associate Professor in Nursing,. She is an experienced International Board-Certified Lactation Consultant (IBCLC), a community-engaged participatory action and reproductive health equity researcher who uses a critical ethnographic lens. All of the authors bring their own lived experiences and understanding of the perspectives of perinatal healthcare providers on respectful maternity care, which may affect our analysis and interpretation of the data.

## Method

This scoping review was conducted in accordance with the guidelines and checklist provided by the Preferred Reporting Items for Systematic Review and Meta-analysis extension for Scoping Reviews (PRISMA-ScR; [39]. The review procedures were also guided by the Joanna Briggs Institute (JBI) enhanced scoping review framework [40]. The protocol for this review has been registered with the Open Science Framework.

### Eligibility criteria

The Joanna Briggs Institute’s enhanced scoping review framework [40], inclusion and exclusion criteria were set a priori to reduce the risk of introducing bias into the review, thereby promoting the validity of findings. The inclusion and exclusion criteria were guided by a consideration of participants, concepts, context, and types of sources of evidence (see Appendix II). This review was limited to studies published from 2013 to 2024.

Studies available in English and partly or wholly based in the US were eligible for inclusion if they examined the perspectives of perinatal healthcare providers in the US who work in facility-based settings on maternity care provision, respectful maternity care, disrespect and abuse, obstetric violence, and perinatal outcomes.

### Data sources and search strategy

A literature search was conducted in the following four databases: PubMed, Embase, Web of Science, and CINAHL. In line with the inclusion criteria, the search was limited to studies conducted in the US among perinatal healthcare providers, full text in the English language, and published in peer-reviewed scientific journals. Searches were conducted on October 24, 2023, and updated on September 30, 2024, with restrictions to published literature between 2013 and 2023. The Centers for Disease Control and Prevention website, WHO Global Health Library, White Ribbon Respectful Maternity Care Repository, Columbia Public Health website, and Google Scholar were searched for additional journal publications. A final updated search was conducted on March 5, 2025, covering the period from 2013 to 2024.

The searches were carried out using a set of predefined medical subject headings (MeSH) as search terms for each database: PubMed, Embase, Web of Science, and CINAHL (see Appendix III). The search strings were created by combining terms such as “healthcare providers,” “clinicians,” “respectful maternity care,” “humanization in birth,” and “compassionate maternity care” with the BOOLEAN operators (“AND”/“OR”). In addition, hand searches of reference lists from studies retrieved in the initial database search were conducted. A medical librarian at the University of California San Francisco was consulted on the search strategies for all databases that were reviewed.

### Study selection

Following the PRISMA-ScR guidelines [39], each article was independently assessed for eligibility. After the final updated search, 1,706 articles were retrieved from PubMed, Embase, Web of Science, and CINAHL databases. The retrieved articles were imported into Zotero reference management software [41], which resulted in the removal of 114 duplicate articles. The remaining 1,592 citations were imported to Covidence web-based computer software (Melbourne, Victoria, Australia) for additional duplicate removal and reference screening. Two independent reviewers conducted title and abstract screening of the remainder 954 citations to obtain articles that met the eligibility criteria for full text review. Nine-hundred and fifteen irrelevant articles were removed. The full text of 39 articles were reviewed by two independent reviewers using the study’s a priori inclusion criteria. Discrepancies during title, abstract, and full-text screening were resolved by discussion with a third reviewer until a consensus was reached. Fourteen studies were eligible for data extraction.

### Data charting and synthesis

The data were collected using a standardized form that included domains such as study setting, sample characteristics, objectives, study design, data collection and analysis methods, and conclusions. The review assessed the extracted data on providers’ perspectives on and experiences of disrespect, abuse, and obstetric violence during the perinatal period, facilitators and barriers to the provision of respectful care, and how providers observe the elements of respectful maternity care in their everyday practice. For the sole quantitative study included in this review, a narrative description of study characteristics, methods, predictors, outcome measures, and major findings is presented.

In addition, a thematic synthesis approach was used to analyze and synthesize the text units extracted from the included studies [42]. The thematic synthesis was informed by existing frameworks on childbearing women’s rights, respectful maternity care, and mistreatment of women typologies [24, 43]. We developed and used an axial coding scheme to sort the initial codes iteratively and underlying data into first-order, second-order, third-order, and fourth-order themes [24, 42, 44]. First-order themes represent primary text units grouped based on common descriptive themes. Second-order themes were created by grouping first-order themes based on higher-level analytic themes. Third-order themes represent second-order themes grouped based on some underlying shared attributes. Fourth-order themes represent overarching high-level analytical themes comprising the first, second, and third-level themes.

### Critical appraisal

Identified studies that met the eligibility criteria were appraised for relevance and rigor using the Mixed Methods Appraisal Tool (MMAT) [45]. Since research on provider perspectives on respectful maternity care in the US is still emerging, the characteristics and variables of the studies are heterogeneous. Therefore, the MMAT is an appropriate appraisal tool for the included studies. The MMAT consists of two screening questions that can be applied to all study designs, followed by five items unique to each study design [45]. The screening questions were used to determine if there are clear research questions and whether the data collected allows us to address the research question [45]. The methodological quality criteria for quantitative non-randomized trials and qualitative designs were used for this scoping review. See Appendix IV for the assessment criteria. Response options for all items are “Yes,” “No,” and “Can’t tell” [45]. These assessment scores were reported as proportions or percentages to ensure standardized comparison across study designs [45].

## Results

### Search results

**Fig. 1:**
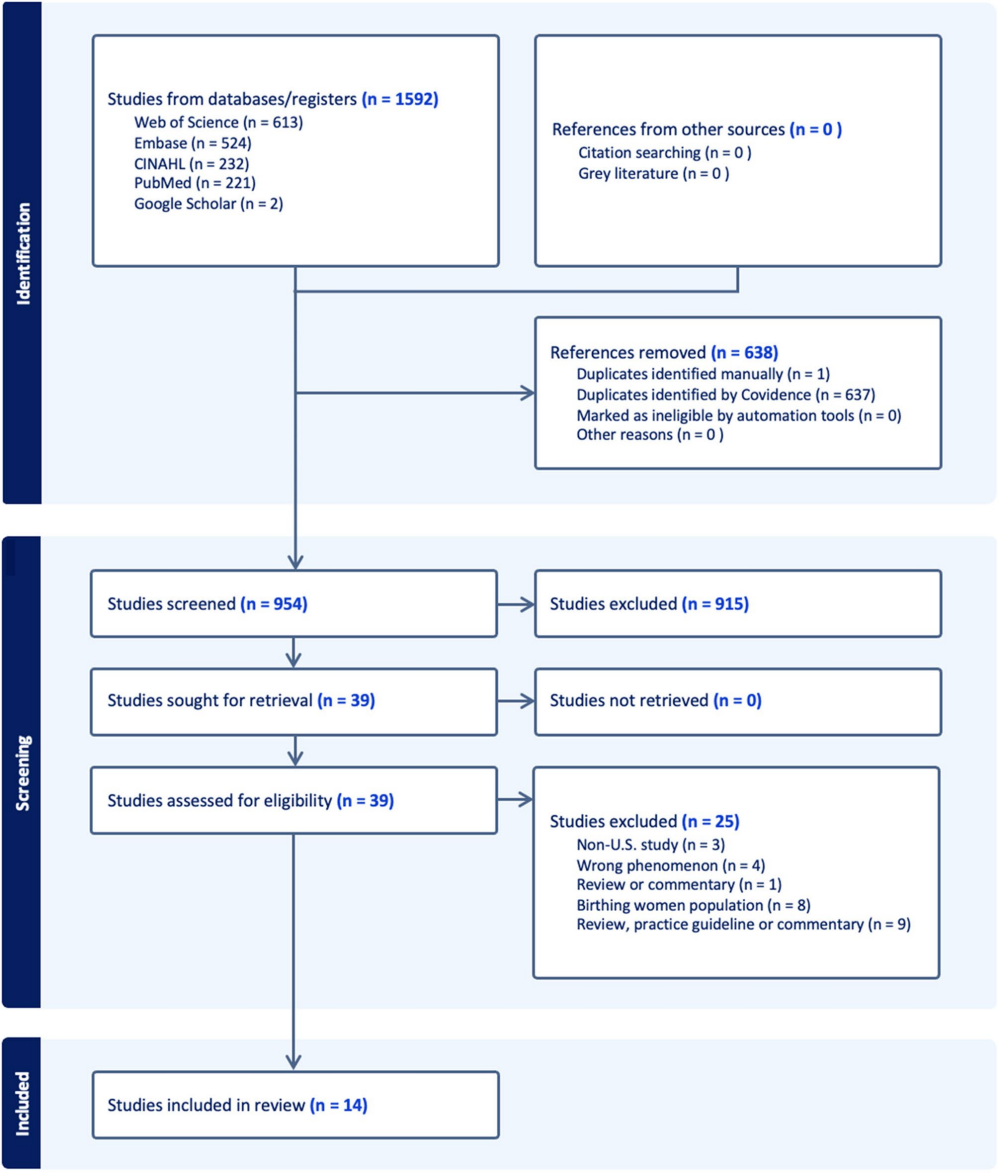
Flow Diagram of the Literature Search

The initial electronic literature searches conducted in PubMed, CINAHL, Web of Science, and Embase yielded 764, and the updated searches yielded an additional 55 articles, yielding 819 articles in total. Before the screening, 191 duplicate records were removed. Title and abstract screening excluded 584 records. Full texts were retrieved for 33 potentially eligible studies. After exclusions, ultimately, 11 studies were included. A PRISMA-ScR flow diagram outlining the study selection process, including reasons for exclusion, can be found in Fig. 1 below.

### Study characteristics

#### Study design & sources of data

This review included two (*n* = 2) quantitative studies [46, 47], one mixed method study [48] and eleven (*n* = 11) qualitative studies [21, 49–58]. One of the quantitative studies employed a cross-sectional design [47], while the other used pretest/posttest design [46]. The mixed method study also employed a cross-sectional online survey and qualitative interviews. Of the 11 qualitative studies, only two reported adherence to a recognized qualitative research design such as grounded theory, ethnography, and phenomenology [49, 51]. The two that reported qualitative research designs used either grounded theory [51] or phenomenology [49]. All the qualitative studies used semi-structured interviews for data collection. Of these, four (4/11, 36.3%) used focused group discussions in addition to the interviews [49, 51, 56, 59]. The qualitative data analysis methods reported include thematic analysis (5/11, 45.5%) [49, 50, 53, 55, 58]; modified grounded theory iterative process and constant comparative method (1/11, 9.1%) [21]; a modified form of grounded theory using inductive and deductive coding (1/11, 9.1) [51]; rapid qualitative approach with inductive coding (1/11, 9.1) [56]; and content analysis (3/11, 27.3%) [52, 54, 57]. For the quantitative studies, data were collected via an online maternity support survey (2012–2013) and a pre/posttest survey [46, 47]. The quantitative data were analyzed using descriptive statistics, logistic regression, and Wilcoxon signed rank test [46, 47]. See Table 1.

**Table 1:** Characteristics of sources of evidence

	First author/ published year	Question(s) or Aim(s) of Study	Sampling & Sample Characteristics	Maternity Care Setting	Study Design	Predictors	Outcome Measures	Patient Population Focus
1	Chambers et al. (2022) [21]	To explore clinician perceptions of how racism affects Black women’s pregnancy experiences, perinatal care, and birth outcomes.	***Sampling***: Convenience ***Characteristics***: Perinatal care providers (*n* = 25), including: • OB/GYN (*n* = 11) • Certified nurse midwives (*n* = 8), • Family medicine practitioners (*n* = 2) • Fellows/residents (*n* = 4) • White providers (*n* = 16, 64%) • Women providers (*n* = 23, 92%).	Two hospital facilities	***Approach***: Qualitative ***Design***: Not stated ***Data Collection***: Semi-structured interviews ***Data Analysis***: Modified grounded theory iterative and constant comparative method.	N/A	N/A	Black women
2	Salter et al. (2023) [54]	To explore whether maternity care providers’ descriptions of patient birth trauma overlap with categories of mistreatment from a globally accepted typology.	***Sampling***: Purposive and snowballing ***Characteristics***: Providers (*n* = 28), including: • Physicians (*n* = 6) • Certified nurse midwives (*n* = 8) • Labor & delivery nurses (*n* = 14)	University-affiliated maternity care hospital, small suburban hospital, and a free-standing birth center.	***Approach*** Qualitative ***Design***: Not stated ***Data Collection*** Descriptive semi-structured interviews ***Data Analysis***: Content analysis	N/A	N/A	General maternity care patient population
3	Morton et al. (2018) [47]	To compare birth doulas’ and labor and delivery nurses’ reports of witnessing disrespectful care in the United States and Canada.	***Sampling***: convenience, through nine professional organizations ***Characteristics***: Maternity support workers in US and Canada (*n* = 2781), including: • Total labor & delivery nurses (*n* = 967) • US L&D nurses (*n* = 840) • Total doulas (*n* = 1435) • U.S doulas (*n* = 1160) • White doulas (*n* = 1338) • White L&D nurses (*n* = 915)	Not stated	***Approach***: Quantitative ***Design***: Cross-sectional ***Data Collection***: Online survey ***Data Analysis*** • Descriptive statistics • Logistic regression	Professional role (L&D nurse vs. doula) age, education, race/ethnicity, household income, marital status, parental status,, work-family conflict, emotional regulation, region, Baby-Friendly Hospital status, and intentions to leave maternity support work within three years	Perceptions of frequency of disrespectful care, including threats that a baby might die, racist remarks, sexist remarks, extra procedures because of race-ethnicity, lack of informed consent, and acting explicitly against a woman’s wishes.	General maternity care population
4	Decker et al. (2021) [51]	To compare the perinatal experiences and perspectives of Mexican-origin adolescents and their healthcare providers in two distinct sociocultural and healthcare contexts.	***Sampling***: Purposive ***Characteristics***: Providers in US and Mexico (*n* = 15), including: • Medical assistants, health educators, nurse practitioners, and youth health program directors from California (*n* = 9) • Physicians, nurses, social workers, and health educators from Mexico (*n* = 6)	Not stated	***Approach***: Qualitative ***Design***: Grounded theory ***Data Collection***: In-depth interviews ***Data Analysis***: Modified grounded theory using deductive and inductive coding, including an iterative and reciprocal data-theory relationship	N/A	N/A	Pregnant Latina adolescents population
5	Smith et al. (2022) [55]	To gather and synthesize the experiences of Black women who delivered prematurely, along with the clinicians and community-based organizations that support them.	***Sampling***: Convenience ***Characteristics***: Black women (*n* = 30) Community-based organizations representatives (*n* = 32) Clinicians (*n* = 19)	Hospitals, community clinics and birth centers	***Approach***: Qualitative ***Design***: Not stated ***Data Collection***: Focused group Key informant interview ***Data Analysis***: Thematic analysis	N/A	N/A	Black birthing women patient population
6	Syvertsen et al. (2021) [57]	To examine how stigma manifested across women’s pregnancy journeys to shape access and quality of care.	***Sampling***: convenience and snowballing ***Characteristics***: Women with histories of opioid misuse who were pregnant or recently gave birth (*n* = 28) Healthcare providers (*n* = 18)	Not stated	***Approach***: Qualitative ***Design***: Not stated ***Data Collection***: Semi-structured interview ***Data Analysis***: Content analysis using cascade of care” framework.	N/A	N/A	General maternity care patient population, predominately White (79%)
7	Reed et al. (2023) [53]	To explore providers’ experiences with and perceptions of how doula presence impacts their work and patient experience.	***Sampling***: convenience and snowballing ***Characteristics***: 28 providers including: Physicians (OB/GYN, family medicine, anesthesiology) (*n* = 11, 39%) Nurses (*n* = 10) Certified nurse-midwives (*n* = 7). • White providers (*n* = 23, 82%) • Latina, Latinx, or Spanish (*n* = 3, 11%). • Asian (*n* = 4, 14%) • American Indian or Alaska Native (*n* = 1) • Women (*n* = 27, 96%)	Hospitals and clinics	***Approach***: Qualitative ***Design***: Not stated ***Data Collection***: In-depth interviews ***Data Analysis***: Modified reflexive thematic analysis.	N/A	N/A	General maternity care patient population
8	Coley et al. (2018) [50]	To compare African-American and mixed-race mothers and prenatal providers’ perceptions of prenatal care quality structures and processes with a focus on patient-provider interactions and person-centered care. To compare perceptions of prenatal care quality among mothers of different socioeconomic backgrounds, using insurance type as an indicator.	***Sampling***: Purposive ***Characteristics***: Prenatal care providers (*n* = 20) White providers (*n* = 18) Mothers who recently gave birth (*n* = 19)	Fourteen clinics, private and academic medical centers, federally qualified health centers offering prenatal care services with individual, group, and midwifery care.	***Approach***: Qualitative ***Design***: Not stated ***Data Collection***: Semi-structured interviews ***Data Analysis***: Thematic analysis	N/A	N/A	Black women maternity care patient population
9	Moniz et al. (2022) [52]	To examine how peripartum contraceptive care quality improvement efforts address or perpetuate reproductive health injustices.	***Sampling***: Purposive (by description, but not stated) ***Characteristics***: Seventy-eight key informants, including: Providers (*n* = 45), Operations staff (*n* = 24), Hospital administrators (*n* = 9).	11 academic medical centers in the United States	***Approach***: Qualitative ***Design***: Not stated ***Data Collection***: In-depth semi-structured interviews ***Data Analysis***: Inductive content analysis	N/A	N/A	General maternity care patient population
10	Spigel et al. (2022) [56]	To examine the facilitators, barriers, attitudes, and contextual fac- tors influencing clinicians’ experience with TeamBirth, a Shared Decision-Making solution that prioritizes the birthing person’s involvement in decision-making.	***Sampling***: Purposive ***Characteristics***: Sampled 103 clinicians across the four L&D units implementing TeamBirth, including: • Nurses (*n* = 58) • Obstetricians (*n* = 37) • Midwives (*n* = 8)	Four high-volume hospitals and medical centers across the United States	***Approach***: Qualitative ***Design***: Not stated ***Data Collection***: In-depth semi-structured interviews and Focused group ***Data Analysis***: Rapid qualitative approach and Inductive coding	N/A	N/A	General maternity care patient population
11	Ayers et al. (2018) [49]	To examine maternal health care providers’ perceptions and experiences of barriers in providing care to Marshallese women and also investigate providers’ perceived barriers of access to care among Marshallese women.	***Sampling***: Purposive and snowball ***Characteristics***: Sampled 20 perinatal health care providers, including: • Nurses (*n* = 8) • Advance Practice Nurses (*n* = 4) • OBGYNs (*n* = 3) • White providers (*n* = 15, 88.2%) • Women providers (*n* = 15, 88.2%). • Five provider demographics not accounted for.	Not stated	***Approach***: Qualitative ***Design***: phenomenology ***Data Collection***: In-depth interviews and Focused group ***Data Analysis***: Thematic coding	N/A	N/A	Marshallese women maternity care patient population
12	Hills et al. (2024) [46]	To measure the impact of an evidence-based guideline on respectful maternity care on nurses’ attitudes and beliefs about childbirth practices	***Sampling***: convenience sample ***Characteristics***: • Registered nurses – (*n* = 9)	An intrapartum unit at a tertiary care center in the south- eastern United States	***Approach***: Quantitative ***Design***: pretest/posttest ***Data Collection***: 42-item Nurse Attitudes and Beliefs Questionnaire–Revised ***Data Analysis*** • Descriptive statistics • Wilcoxon signed rank test	The “Respectful Maternity Care Framework and Evidence- Based Clinical Practice Guideline” (RMC EBG) was implemented as a routine standard of practice in an intrapartum unit.	Lower scores are reflective of attitudes and beliefs that support a medical model of care, whereas higher scores are reflective of a physiologic model of care.	Not Stated
13	Snyder, (2024) [58]	What types of harm are doulas and midwives witnessing? What factors influence their decision to intervene? What impact does this experience have on their professional career and well-being?	***Sampling***: Purposive and snowball ***Characteristics***: Sampled 17 doulas and midwives	Not stated	***Approach***: Qualitative ***Design***: ***Data Collection***: Semi-structured qualitative interviews ***Data Analysis*** Thematic analysis based on Braun and Clarke’s updated reflexive method	N/A	N/A	Not stated
14	Fachon et al. (2024) [48]	To examine obstetric care providers’ observation and perception of underlying root causes of disrespect and abuse at an urban tertiary care center in the USA	***Sampling***: convenience sample ***Characteristics***: Sampled 46 care team members on the labor and delivery floor Nurse (*n* = 23, 50.0%) Midwife (*n* = 8, 17.4%) Physician (*n* = 15, 32.6%)	Labor and delivery floor of Massachusetts General Hospital (MGH) in Boston	***Approach*** Mixed-method ***Design***: Cross-sectional design ***Data Collection*** Online survey ***Data Analysis*** Descriptive statistics of proportions and frequencies of variables. Exploratory analysis examined the difference in proportions between different occupations (physicians, midwives, and nurses) using Fisher’s exact test.	Not stated	Not stated	Not stated

#### Conceptual framework

In this review, 50% (7/14) of the included studies reported using a conceptual or theoretical framework [46, 47, 50, 54, 56–58], while 50% of them did not [21, 48, 49, 51–53, 55] (Table 2). The frameworks included Bohren et al.’s [24] Seven-Item Typology of Disrespectful Care during childbirth [47, 54], the Multidimensional Framework of Stigma [57], Donabedian’s Model of Health Care Quality [50], Feminist Stand Point Theory [58], the Consolidated Framework for Implementation Research [56], AWHONN, (2022) Respectful Maternity Care Framework and Evidence- Based Clinical Practice Guideline [46], and Socio-Ecological Model [51]. While six of the studies used a theoretical or conceptual framework to inform the study design, data collection, and analysis process, one study only discussed the socio-ecological model in relation to data coding [51], another study also utilized the theory as intervention measure for quality improvement [46].

**Table 2:** Key findings

	First author/ published year	Theoretical/Conceptual Framework	Measures Used to Ensure Validity/Rigor/Trustworthiness	Major Findings: Providers Perspectives of Perinatal Care
1	Chambers et al., 2022 [21]	Not stated	The study was ethically approved. Debriefing with research team, Using field notes Two independent coders with an inter-rater reliability of 93%	Participants expressed a heightened awareness of structural racism and how it impacts Black women’s health outcomes, medical education, and providers’ interactions with patients. Racism influences providers’ ability to acknowledge and engage Black women as agents of their bodies and the care provided by hospitals. Racism influences providers’ perceptions of Black women, children, and families, resulting in punitive care and treatment such as unwarranted urine toxicology (UTOX) screening or child protective services (CPS) involvement. Providers acknowledged that the field of obstetrics and gynecology has deep-seated racist ideologies and behaviors that must be dismantled to improve the reproductive health experiences and outcomes of Black women. Providers struggle to identify how racism impacted their own care practices, although they acknowledge the presence and consequence of structural racism on Black maternity care experience and outcomes. Policies and procedures must be implemented to hold providers accountable for providing equitable and respectful care to Black women, children, and families. Providers observed the need to implement policies and procedures to hold providers accountable for providing equitable and respectful care to Black women, children, and families.
2	Salter et al., 2023 [54]	Bohren et al.’s (2015) seven-item typology of disrespectful care during childbirth	The study was ethically approved. Engaging community partners, field testing interview guide, and incorporating feedback. Use of direct participant quotes.	Overall, some healthcare providers may use the phrase “birth trauma” as a euphemism to describe mistreatment. Providers’ descriptive narratives of birth mirrored patient descriptions and experiences of injustice. About 25% of participants described having experienced situations where colleagues did not introduce themselves to patients, perform cervical exams, cut episiotomies, rupture membranes, and perform vacuum-assisted deliveries without obtaining consent from the patient. 21.4% of participants narrated that patients were stigmatized and discriminated against based on their social characteristics. Participants pointed to hospital policies having differential effects on some groups of patients. For example, patients of color or Latinx ethnicity are mandated to drug screening if they miss multiple prenatal visits. Minority and low SES patients who miss scheduled appointment don’t need drug screens; they need transportation. Hospital visitor policy, not always culturally acceptable to. Black families that tend to want … more of their family members there. Two midwives, one nurse, a physician observed verbal abuse (i.e., scolding or yelling, judgmental or accusatory comments, threats to withholding treatment, and threats of poor outcomes, and blaming patients for poor outcomes). An obstetrician related a story in which an anesthesiologist screamed at a woman, told her she would not receive an epidural if she did not stop moving, and then left the patient’s room. `Statements from six different providers (a midwife, two nurses, and three physicians) show ineffective comunication, including dismissal of birthing people’s concerns and poor maternity care staff attitudes that show lack of supportive care and violate women’s autonomy. Physical abuse: One nurse’s narrated how a *charge nurse … pushed her out of the way and grabbed a laboring woman’s leg*,* and was in her face and screaming*,* “You’re not pushing …”* One nurse described her own behavior threatening a woman of color that if she leaves the hospital against the medical advice, “your baby’s going to die,” saying that sometimes, one needs to be a little rough: *‘… We’re not really supposed to*,* but sometimes that’s what you got to tell them … “If you do this*,* if you leave against our medical advice*,* your baby’s going to die”’*
3	Morton et al. (2018) [47]	Bohren et al.’s (2015) seven-item typology of disrespectful care during childbirth	The study was ethically approved, but the survey tool or instrument used to collect data on participant experience of disrespect and abuse was not stated. The internal consistency reliability of the emotional regulation subscale was computed (α = 0.84).	A failure to meet professional standards was the most common form of disrespectful care reported by the respondent. Overall, 65.4% of all participants reported they had witnessed providers occasionally or often engaging in procedures without giving a woman the time or option to consider them. One-third of the providers reported verbal abuse in the form of threats to the baby’s life. Doulas are significantly more likely than nurses to report verbal abuse during childbirth. Experiencing disrespectful care increased likelihood of leaving maternity support work within three years. 18% of respondents witnessed providers performing procedures against patient wishes—doulas reported 8.5% more than nurses. Doulas of color had 3.3 times higher odds of reporting that they occasionally or often heard care providers make racially demeaning comments and 2.4 times higher odds of witnessing care providers perform extra procedures based on a woman’s race or ethnicity compared to White, non-Hispanic providers. Nurses at (aspiring) Baby-Friendly hospitals reported less often that they witnessed providers performing procedures explicitly against a woman’s wishes.
4	Decker et al. (2021) [51]		Weekly meetings were held to review coding, clarify codes, and modify the codebook for improved reliability. Inter-coder reliability was tested to ensure coding consistency, resulting in a Cohen’s kappa value of 0.88. The use of direct participants quotes.	Providers in Guanajuato, Mexico and California locations identified several challenges communicating effectively with their adolescent patients, including personal opinions about adolescent pregnancy and structural issues. Some providers are aware of patients’ criticisms around lack of respectful, patient-centered care. Providers often attributed their inability to provide patient-centered care to structural constraints. Some also recognize their own negative opinions about their adolescent patients’ life choices and pregnancy. Providers found it challenging to provide care when they strongly disagreed with their adolescent patients’ life choices and pregnancy. Unlike patients, providers were more likely to focus on structural challenges such as limited appointment times, institutional support, and insurance and other documentation issues. Providers in both countries expressed concern about patient caseload and the resulting limits on how much time they could spend per patient. Young women feel uncomfortable speaking directly with their doctors. One medical assistant in California discussed how they facilitate communication between doctors and adolescents. In California, many providers described language barriers when communicating with immigrant youth and their families, particularly when needing to convey complex information. Several providers in California also mentioned that concerns about insurance and immigration status limited interactions with patients. Providers in California discussed the power imbalance between providers and adolescent patients. The providers reported that many youths do not feel empowered to make their choices known, particularly during the time of delivery, or that their opinions are ignored by medical personnel. Healthcare providers in California raised concerns about the negative impact of mandated reporting laws, which require them to report any sexual activity between an adult and a minor. This law hinders communication between the patient and the provider.
5	Smith et al. (2022) [55]	Not stated	The study was ethically approved. Compare coding schemes to identify agreements and resolve disagreements between the analysis team. Use of direct participants’ quotes	Providers reported that they don’t have enough time to spend with their patients, hence unable to build rapport with them. A broken healthcare system makes it difficult for patients to trust us. Patients find it hard to accept advice and interventions due to previous bad experiences. They believe they will have another bad experience. The providers noted that the lack of emphasis on prevention within the healthcare, which leads to missed opportunities that allow for racial inequities in birth outcomes to persist. A physician reported that they sometimes don’t listen to mothers because when they make plans, mothers don’t adhere to them. Providers rely on heuristics from their training and past clinical experiences to address patient needs. However, implicit biases and limited time keep providers from closely addressing the needs of Black women. Medical training doesn’t emphasize listening. It is not unique to African American women to feel unheard, and this widespread complaint in healthcare profession and points to the need to learn to work better with individuals. All participants agreed that providing culturally congruent care leads to improved perinatal outcomes.
6	Syvertsen et al. (2021) [57[	The multidimensional framework of stigma		Providers narrated that some obstetricians “fire” pregnant patients found using drugs. Providers’ liability concerns have made it difficult for individuals to access concurrent prenatal care drug treatment. Obstetricians are often hesitant to treat women with addiction issues, and addiction specialists may be hesitant to treat pregnant women. Structural stigma hinders women’s access to drug treatment due to policies that treat drug use as an exceptional condition. Healthcare providers recognize that stigma is a significant problem across healthcare settings. Staff struggle with their own biases. Pregnant women rightly read non-verbal cues about providers’ judgmental behaviors and attitudes. Stigma in mental health treatment is perpetuated by insurance billing practices, high costs, and long waitlists. Recognizing the resiliency of pregnant women who use opioids is crucial in changing provider attitudes towards them, especially considering the challenging contexts in which they live.
7	Reed et al. (2023) [53]	Not stated	The study was ethically approved. Discuss reflections about the data and identify potential themes. wrote reflexive memos. The use of direct participants quotes.	Overall, participants reported that doula presence positively impacted healthcare providers’ experience of providing pregnancy-related care and their perception of patients’ care experience. Providers reported that, doula presence increased providers’ personal sense of accountability toward their patients. Doula presence increased institutional-level accountability. Participants perceived doulas to improve the experience of care for pregnant and birthing people by providing services, such as continuous labor support, which providers are unable to deliver due to the limitations of their roles. Doulas’ presence allows medical professionals to focus on clinical tasks. Doulas make providers’ work easier by providing continuous support to clients. Participants narrated that having a doula present increased their sense of accountability when interacting with patients. With doulas presence, providers were more conscious about how they communicated with patients and approached care. Participants perceived patients with doulas to be more informed; hence, they were inclined to communicate details to those patients about their care. Participants reported that doulas act as advocates for clients, empowering them to self-advocate and reshaping power dynamics in the patient-provider relationship. Most participants believe institutional racism and implicit bias impact care quality. Implicit bias influences providers’ assumptions about what patients might want, influences the direction of care, and limits options provided to patients. It can be challenging to detect instances of racism and bias and to hold colleagues responsible for such behavior. Some nurses, primarily white, indicated that they do not believe implicit bias or institutional racism impacts pregnancy-related care. Most of the nurses who believe there’s not implicit bias or institutional racism also strongly expressed that doulas can negatively impact their work by creating mistrust of the hospital staff and questioning providers’ recommended interventions. Providers reported that they are typically unable to provide continuous labor support to patients despite its benefits due to simultaneously managing clinical tasks among multiple patients.
8	Coley et al. (2018) [50]	Donabedian’s (1988) model of health care quality	Two members coded each transcript. Inter-rater reliability was checked using percentage agreement of themes. Consensus meetings were held to discuss and resolve coding discrepancies. Audit trail of coding decisions was kept throughout the analysis process. Emergent themes were mapped onto Donabedian’s (1988) conceptual model of health care quality to ensure relevance of results to current literature. Institutional review board approved.	In contrast to mothers’ perceptions of prenatal care quality centered around interpersonal processes during hospital visits, providers perceptions of prenatal care quality focused more on activities that constitute “quality prenatal care” based on American College of Obstetrics and Gynecology (ACOG) standards such as completion of required tests and communicating this information to the patient. Providers highly valued care characteristics that are indicative of a patient-centered approach. This includes providing compassionate care that meets the needs of mothers, establishing a functional mother-provider relationship, effectively responding to patient questions, providing prenatal education to ensure well-informed mothers and offering care that looks at the overall wellbeing of the woman. Mothers’ satisfaction with experienced care is a key outcome of quality prenatal care, according to providers. Several providers emphasized the importance of being satisfied with the care they provide. Some providers held skeptical views about the importance of cultural competency and sensitivity for the quality of prenatal care they are able to offer women. Providers viewed access to good clinic teams as important aspects of prenatal care quality.
9	Moniz et al. (2022) [52]	Not stated	Strategies to enhance credibility and trust- worthiness of findings included constant comparison, consensus coding, illuminating findings with participants’ own language via quotes, and critical reflection about researchers’ personal biases.	Providers identified respectful care as a core value; described their contraceptive counseling practices as based on group decision-making. There is a growing concern in the family planning community that excessive promotion of long-acting reversible contraceptives could limit patient autonomy. Providers described their struggles with how best to ensure access to long-acting contraceptives while ensuring patients do not experience those efforts as a coercive promotion. Providers often face a conflict between their own desire to prioritize their patients’ needs and preferences, and the clinical policies and practices that fail to put patients at the center. Othering language used to describe groups different from providers’ own or favored groups. Broad generalizations and stereotyping based on patient socio-demographics. Evidence of biased language that invoke stereotypes was frequently used by providers who believed that immediate postpartum long-acting contraceptives were appropriate for some patients but not for others. Providers recognize structural inequities in the provision of postpartum contraceptive care. Example: The institutional policy of offering long-acting reversible contraceptives (LARC) only to Medicaid enrollees allowed healthcare workers to act on biased assumptions about who should or should not have more children.
10	Spigel et al. (2022) [56]	Consolidated Framework for Implementation Research	Consensus coding—three coders, 10% double coded, disagreements resolved through discussions. Institutional review board approved.	Providers primarily valued TeamBirth for promoting clarity about the care plan due to transparent communication and documentation of the care plan on the planning board, leading to full team participation and shared decision-making. Respondents also noted that the intervention promoted a better environment for teamwork by creating space for “sharing control” and evening out the playing field between nurses, doctors, and birthing people. Internal and external contextual factors influenced the accept- ability and feasibility of TeamBirth. Internal factors include leadership commitment, regular and transparent feedback loops, local adaptation, health facility structural characteristics such as nursing ratios and staffing shortages, culture of teamwork and psychological safety, compatibility of TeamBirth messaging with providers’ norms and values such as around reducing unnecessary cesarean births vs. messaging that says TeamBirth enhances patient experience, perceived ease of integrating into existing workflows. For factors external to the hospitals, relationship-building and mentorship between implementation teams across hospitals were seen as important for scaling the shared decision-making intervention. Some observed that positive media coverage helped to gain the support and acceptance of providers, hospital leadership, and patients. In some settings, participation in TeamBirth was motivated by anticipation of external policies such as reimbursement changes among insurance companies.
11	Ayers et al. (2018) [49]	Not stated	To establish reliability, two members coded transcript and one confirmation coder.	Providers described two structural barriers Marshallese patients faced. These barriers include transportation difficulties that prevent patients from attending appointments and a lack of health insurance, which leads to delays in prenatal care initiation and limits their birth control options. Most providers reported communication and language as primary barriers to delivering quality perinatal health care. Providers identified stoicism as a barrier due to difficulty reading nonverbal communication among Marshallese patients. Providers reported their admiration for individual patients they perceived had a high pain tolerance. Also, providers identified their inability to interpret Marshallese patients’ emotional affects as a barrier. Due to this, they are unable to read women’s physical pain during birth, leaving them uncertain about when the baby is coming. Providers reported difficulty establishing trust and communication with Marshallese patients. Providers described patients as giving brief responses and not asking questions, constraining their ability to provide care. Providers’ perceptions of the Marshallese community’s collectivist culture is at odds with the individualistic culture in America. Lack of understanding of the collectivist, supportive nature of the Marshallese community is challenging to providers in regard to hospital protocol or typical procedures. Providers often interpreted large number of family members as inappropriate rather than supportive. Judgmental discourse used when discussing the collectivist nature of the Marshallese community: “It’s like is this a celebration of the birth or did they have no other place to be?” “Is this an opportunity for them to have a mini-vacation and go to the hospital and have an air-conditioned place to stay?” The notion that some Marshallese communities resist adopting the western models of perinatal healthcare. Much of the discourse surrounding the lack of early and consistent perinatal healthcare among Marshallese patients has often attributed the issue to a lack of patient prioritization, rather than to the intersection of numerous dynamic structural and communication barriers.
12	Hill et al. (2024) [46]	Respectful Maternity Care Framework and Evidence- Based Clinical Practice Guideline” (RMC EBG; AWHONN, 2022)	This project and was approved under federal regulations (45CFR46.110) by the Office of Research at the participating clinical site. Additionally, institutional review board approval for the project was granted by the participating university. To protect the data, the surveys were created in Survey Monkey with a password-protected organizational account owned by AWHONN.	Although there was no change in nurse attitude and beliefs about childbirth practices after 3 months (p 1⁄4 0.058), the aggregate scores on a scale of 42 to 168 increased by 5.6 points. Two subscales of the Nurse Attitudes and Beliefs Questionnaire–Revised—Medical Model of Conflict and Women’s Autonomy—had the greatest increase in aggregate scores.
13	Snyder, (2024) [58]	Feminist standpoint theory	Author was aware of her positionality and engaged in reflexivity, as well as peer and mentor debrief	While the language around obstetric violence is a topic of debate, yet participants expressed their unique perspectives on their experiences in the delivery room. They recounted various detrimental practices they witnessed 14during labor and childbirth with every participant discussing a spectrum of violence they encountered. A participant shared: “*Oftentimes*,* medical professionals will enter the room and not introduce themselves and just come to the bedside and stand over the woman with this energy that exerts the idea that they are the doctor*,* they know what is best*,* and if you want to give birth in their hospital*,* on their watch*,* you will do what they say.* “ Participants also described witnessing experiences that they classified as “outright assault.” Determining when and how to step in for a client facing a type of obstetric violence was a complex issue for many participants. At times, there wasn’t sufficient time to intervene because events were unfolding rapidly. Additionally, as Amaya noted, there was a concern about “fear of retaliation” and a feeling of being “uncertain about what my client wanted me to do.” Most participants described this moment of decision-making as difficult. When questioned about this, doula Sara commented: *“You always have to calculate the risk of speaking up. You are constantly gauging what to do*,* and some- times*,* you have a matter of seconds to figure it out. You want to speak up when you see something that is not in alignment with what your client wants*,* but you always run the risk of retaliation*,* being removed from the room*,* or being ignored. It is hard to figure out what to do”*
14	Fachon et al. (2024) [48]	Not stated	The study protocol was approved by the Mass General Brigham Human Research Committee. Data were collected through an open and anonymous voluntary electronic survey To ensure confidentiality, the data collection tool was available as an open survey. Participants received a written statement of the research study’s aims and risks before voluntary completion of the online survey. The survey was pilot-tested by a physician, a nurse, and a midwife to check for clarity.	A large majority (84.8%) believed that disrespect and abuse are issues in the obstetrics, and a smaller majority (67.4%) believed it was hospital specific issue. “Disrespect and abuse” was a more familiar term than “obstetric violence”, although both terms were recognized by most respondents. Dismissing patients’ pain was the most (87.0%), reported disrespectful behavior observed followed by discriminatory care based on physical characteristics (67.4%) and race (65.2%), and uncomfortable vaginal examinations (65.2%). Qualitative responses further elaborated on the dismissal of pain or other concerns, one noting they had witnessed “Dismissing concerns about fetal status, nausea, anxiety”. In comparison to other forms of disrespect in medical settings, unwarranted procedures or those done without proper explanation were reported less often. The most frequently cited problematic procedures included artificial rupture of membranes (39.1% experienced or heard of it being done without explanation), followed by episiotomy and membrane stripping (both at 34.8%). Less than 20% of respondents reported witnessing unexplained procedures like Cesarean sections, suturing, blood transfusions, sterilizations, injections, shaving, or catheter placements. Additionally, neglect was a noticeable issue, with over half of the respondents noting instances of patient neglect (52.2%) or leaving patients unattended (54.3%).

#### Study purpose and phenomenon of interest

Overall, all Fourteen studies in this review focused on providers’ perspectives on an aspect of individuals’ perinatal care experience. Of these 14 studies, a majority (11, 78.6%) exclusively explored providers perspectives. The remainder (4, 28.6%) explored perspectives of both providers and birthing individuals [50, 51, 55, 57], with one exclusively focusing on adolescents [51]. One of the fourteen studies examined the attitudinal and contextual factors that influence providers’ experience with and adoption of a shared decision-making intervention that prioritizes women’s involvement in decision-making during childbirth [56]. Another measured the impact of an evidence-based guideline on respectful maternity care on nurses’ attitudes and beliefs about childbirth practices [46]. Additionally, one study also explored providers’ perceptions and experiences of barriers in providing care to Marshallese women and also investigate their perceived barriers of access to care among these women [49]. Of the 14 studies included in this review, three (21.4%) solely explored providers’ perceptions of *Black women’s* maternity care experiences. The three studies examined how racism affects Black women’s perinatal care experience [21], prenatal care quality structures and processes with a focus on patient-provider interactions and person-centered care [50], and the experience of providers who care for Black women who delivered prematurely [55]. One of the study also examined obstetric care providers’ observation and perception of underlying root causes of disrespect and abuse. However, none of the 14 studies focused on providers admitting to their own practices that are or could be deemed disrespectful to pregnant people, particularly Black individuals. Instead, they reported on providers observing and reporting on the behaviors of other providers.

#### Study setting

Per the inclusion criteria, all the studies in this review collected some or all of the data in the US (Table 1). However, only data on US providers are included in this scoping review. Of the 14 studies included in this review, twelve of them were based solely in the US [21, 46, 48–50, 52, 53, 55–58]. Two studies were binational: the US and Mexico [51] and the US and Canada [47]. Only result from the US sample were included in this review. The data collection in the US took place in California (4/14, 28.6%) [21, 51, 53, 55], Mid-western US (3/14, 21.4%) [50, 54, 57], Eastern region (1/14, 7%), and two took place in the Southern region of the US (2/14, 9%). Four studies did not specify where the data were collected [47, 52, 56, 58]. Nine of the studies in this review included perinatal care providers recruited from medical centers and hospitals [21, 46, 48, 50, 53–55] and clinics and birth centers [50, 53, 55]. 36% (5/14) of the included studies did not specify the care setting of the providers recruited.

#### Sampling methods and sample characteristics

Of the fourteen studies, half used purposive sampling (7/14, 50%) [49–52, 54, 56, 58], and half used convenience sampling (7/14, 50%) [21, 46–48, 53, 55, 57]. Three of the 14 included studies combined purposive with snowball sampling [49, 54, 58]. The other two combined convenience sampling with snowballing [53, 57]. The average sample size for maternity care providers in the eleven qualitative studies included in this review was 30.1, ranging from 15 [51] to 103 [56]. Of the eleven qualitative studies, a majority (7/11, 63.6%) reported sample sizes 20 or greater [21, 49, 50, 52–54, 56]. The two quantitative studies had sample size of 2,781 [47] and nine [46] providers. See Table 1.

While a majority of the studies (11/14, 78.6%) focused exclusively on perinatal care providers such as physicians, nurses, certified nurse midwives, other advanced practice nurses, and doulas, three of the study samples included providers as well as Black pregnant women and community-based organization representatives [55], women with histories of opioid misuse who were pregnant or recently gave birth [57] and operations staff and hospital administrators [52]. Six of the studies (43%) did not report a breakdown of the provider professional roles included in the samples drawn [50–52, 55, 57, 58]. Of the eight studies that reported provider roles, 88% (5/8) included physicians, nurses, and nurse-midwives perspectives, and one included doulas’ perspectives [47]. Overall, although doulas are not clinicians, they are the most represented provider category (*n* = 1435), followed by nurses (*n* = 1,057), physicians (*n* = 74), and nurse-midwives (*n* = 35). Although only two studies included doulas in its sample, doulas are the largest group due to the overall large sample size of the quantitative study that included doulas [47]. Of the 14 studies only five of them reported the gender identification of the providers, of which 89.8% identified as female [21, 46, 48, 49, 53]. Among the seven included studies that reported the race/ethnicity of the providers sampled, 81% of the providers were White [21, 46–50, 53].

### Quantitative synthesis

Three studies provided pertinent quantitative data on the effectiveness of a respectful maternity care intervention [46] and providers’ perceptions about disrespect and abuse [47, 48]. Overall, the two studies on providers’ reported observation of disrespectful behaviors include dismissing patients’ pain, discriminatory care based on physical characteristics and race, neglect, coercive or unconsented care [47, 48]. These studies, however, varied in which disrespectful behaviors were reported the most by providers. One found that providers identified failure to meet professional standards such as engaging in procedures like Cesarean sections, suturing, blood transfusions, sterilizations, injections, shaving, or catheter placements without consent, presenting options or against a woman’s wish as the most common form of disrespectful care [47]. Specifically, 65.4% of all participants reporting that they had witnessed providers occasionally or often engaging in procedures without giving a woman the time or options to consider them [47]. The other study found dismissing patients’ pain to be the most reported (87.0%) disrespectful behavior followed by discriminatory care based on physical characteristics (67.4%), race (65.2%), and uncomfortable vaginal examinations (65.2%) [48].

Between 18 and 20% of nurses, midwives, or physicians had witnessed other care providers performing procedures against the patients’ wishes in both studies [47, 48], with doulas reporting 8.5% more than nurses [47]. Additionally, nurses and doulas of color were found to have 3.3 times higher odds of reporting that they occasionally or often heard care providers make racially demeaning comments and 2.4 times higher odds of witnessing care providers perform extra procedures based on a woman’s race or ethnicity compared to White, non-Hispanic providers [47]. Despite providers’ observations about the prevalence of disrespect and abuse in maternity care, the pre-test/post-test study of the effectiveness of a three-month respectful maternity care intervention on a small sample of nurses (*n* = 9) in an intrapartum unit had a slight positive but insignificant effect on nurses’ beliefs about childbirth practices [46].

### Qualitative evidence synthesis

Thematic synthesis of the qualitative data from the twelve studies in this review yielded a six-component typology of perinatal care providers’ views and experiences on respectful maternity care in the United States. The themes were [1] being free from harm and mistreatment [2], rapport between providers and women [3], meeting professional standards of care [4], providing equitable maternity care [5], health facility and system constraints and facilitators, and [6] macro-level, external constraints and facilitators. See Table 3.

**Table 3:** Thematic evidence synthesis of provider perspectives on respectful maternity care barriers and facilitators

FOURTH-ORDER THEMES	THIRD-ORDER THEMES	SECOND-ORDER THEMES	FIRST-ORDER THEMES	CONTRIBUTING STUDIES
*BEING FREE FROM HARM AND MISTREATMENT* • Not using force, shouting and screaming at women • Providing safe, supportive and secure care	**Physical and Verbal Abuse**	Physical abuse	• Use of force	[54]
		Verbal abuse: harsh, threatening, and blaming language	• Racially demeaning comments • Threats to babies’ lives— baby will die • Verbal abuse • Sexually degrading language • Unwarranted procedures (based on race) • Procedures against women’s wishes • Yelling/scolding • Threatening to withhold care • Blaming women of deficiencies in perinatal care • Threats of poor outcomes • Accusatory comments	[47, 49, 54, 55]
*RAPPORT BETWEEN PROVIDERS AND WOMEN* • Preserving women’s dignity and autonomy through cultural competency and sensitivity of the women, and continuous access to family and community support • Engaging with effective communication • Respecting women’s choices that strengthen their capabilities to give birth through addressing provider–patient power imbalance; encouraging active participation and shared decision- making; ensuring freedom of choice, comfort, and providing encouragement; and prioritizing the needs and preferences of women	**#1: Preserving women’s dignity and autonomy** • Cultural Competency and Sensitivity • Family Attendance and Presence of Labor Companions of Choice	Cultural Competency and Sensitivity	• Skeptical views about cultural competency • Lack of cultural sensitivity and competency • Negative attitude toward having many family members • Use of condescending language to describe family/community support practices	[49, 50, 55]
		Family Attendance and Presence of Labor Companions of Choice	• Staff attitudes toward family members • Interpreting having many family members as inappropriate	[49]
	**#2. Engaging with effective communication** • Ineffective Communication • Discomfort Communicating • Trust and Accountability • Language and Interpretation Issues	Ineffective Communication	• Colleagues did not introduce themselves to patients • The need for clear explanations • Dismissing women’s concerns • Personal biases and opinions as a communication constraint • Lack of supportive care • Patients are described as providing brief responses, a constraint on quality care	[49, 51, 54]
		Discomfort Communicating	• Adolescents feeling uncomfortable speaking directly with their doctors • Nurses as facilitating communication between doctors and adolescents	[49, 51, 53, 55, 56]
		Trust and Accountability	• Lack of trust • Difficulty establishing trust and communication • Transparent communication • Providers are more conscious about how they communicated with patients and approached care when doulas are present. • Patients with doulas perceived as more informed • Doula presence increase sense of accountability in provider–patient interactions	
		Language and Interpretation Issues	• Language barriers • Language barriers when communicating with immigrant youth • Inability to interpret culturally diverse patients’ emotional affects•	[49, 51] [53]
	**#4. Respecting Women’s Choices that Strengthen their Capabilities to Give Birth** • Addressing provider–patient Power Imbalance • Encouraging active participation and shared decision making	Provider–patient Power Dynamics	• Adolescents do not feel empowered to make their choices known during childbirth • Power imbalance between providers and patients • Doulas as advocates • Doulas as creating mistrust, leading to questioning, with negative impact on maternity care	[51, 53, 56]
		Shared Decision Making and Patient Autonomy	• Adolescent pregnant women feel their opinions are ignored • Ignoring women concerns and opinions • Not listening to women • Prioritizing patient needs and preferences • Mandatory reporting laws impact trust and care delivery • Conflict between provider desires to prioritize patients’ needs and vs. clinical policies and practices • Implementing shared decision-making interventions • Concern about excessive promotion of apparently beneficial interventions limit patient autonomy • Struggles to ensure recommendations are not experienced as coercive promotion • Respectful care and shared decision-making.as core values	[49, 51, 52, 55–57]
*MEETING PROFESSIONAL STANDARDS OF CARE* • Prospective provision of information and seeking informed consent • Provision of efficient and effective professional care	**#1. Prospective Provision of Information and Seeking Informed Consent**	Unconsented Procedures	• Performing procedures against patient wishes • Not giving enough time to decide • Performing labor and childbirth procedures without asking permission	[47, 54]
		Lack of Information about Treatment Options	• Not giving women treatment options • Failure to meet professional standards as the most common form of disrespectful care	[47]
	**#2. Provision of Efficient and Effective Professional Care**	Biomedical View of Care	• Care focus on running tests • Quality care focused on completing required tests	[50]
		Compassionate Care as an Aspiration	• Awareness of patient criticism of lacking patient-centered humanized care • Birth trauma as a euphemism for mistreatment during childbirth • Patient-centered approach seen as highly valuable • Compassionate care: meets the needs of mothers, establishes a functional mother-provider relationship, responds effectively to patient questions, provides prenatal education to ensure well-informed mothers and offers care that looks at the overall wellbeing of the woman.	[50, 51, 54]
		Pain Relief	• Racialized perception of pain • Pain management and perception	[49]
*HEALTH FACILITY AND SYSTEM CONSTRAINTS AND FACILITATORS* • Quality of Physical Environment, Resources, and Policy, including health facility features such as Baby-Friendly Hospital status, visitor policies, and mandated drug testing policy, and healthcare system culture such as insurance and billing practices, and long waitlists • Continuity of care and professional labor support by providers and doulas • Availability of competent and motivated human resources	**#1: Quality of Physical Environment**,** Resources**,** and Policy**	Hospital Policies	• Mandated drug testing policy for minority groups and low-socioeconomic status groups • Hospital visitor policies • Hospital visitor policy, not always culturally acceptable to Black and Pacific Islander families • Baby-Friendly Hospital designation • Provider–Patient ratio and case load • Policy of offering long-acting reversible contraceptives only to Medicaid enrollees • Policies and procedures to hold providers accountable for inequitable care	[21, 47, 49, 52, 54]
		Healthcare System Culture and Practices	• Liability concerns • Anticipation of external policies such as reimbursement changes among insurance companies • Documentation issues • Limited appointment times • Insurance billing as perpetuating bias • High cost as perpetuating bias • Long wait list as perpetuating bias • Institutional support • Not having enough time to spend with patients • Healthcare system’s lack of emphasis on prevention • Financial need to see a lot of patients in a day • Generally widespread complaint by pregnant women of all races that providers do not listen	[51, 55–57]
	**#2. Availability of Competent and Motivated Human Resources**	Competent and Adequate Staffing	• Patient **caseload** limits how much time providers could spend per patient. • Long waitlist • Feeling that clinician’s training does not adequately prepare them to offer supportive care	[51, 56, 57]
		Professional Training	• Training to cope with patients not adhering to plans • Medical training doesn’t emphasize listening	[55]
		Doula Support and Advocacy	• Doula presence associated with quality of care • Doulas’ presence allows nurses to focus on clinical tasks, affect perception of care	[53]
		Past Experience with Care	• Importance of patient satisfaction with care • Importance of providers satisfaction with care • Bad experiences limits willingness to accept advice and interventions	[50, 55]
		Teamwork and Leadership	• Leadership commitment and culture of teamwork • Perceived ease of new systems	[50, 56]
	**#3. Continuity of Care and Professional Labor Support by Clinicians and Doulas** • Inability to simultaneously manage clinical tasks and continuous labor support Continuous presence of doula staff offers throughout labor and childbirth	Clinical Demands Overwhelm Labor Support	• Demand of clinical role makes it impossible to offer labor support	[53]
		Doulas Offer Continuity of Care	• Doula’s offer labor support, ensuring continuity of care • Doulas make providers’ work easier	
*PROVIDING EQUITABLE MATERNITY CARE* • Stigma and Discrimination (or the absence) based on age, race/ethnicity, medical conditions, or other subgroups • Interpersonal, institutional, and structural racism	**Stigma and Discrimination**	Discrimination Based on Age	• Impact of negative opinions about pregnant adolescent patients on care quality	[51]
		Discrimination Based on Medical Condition (addiction)	• Liability concerns about treating women with history of addition • Bias and structural stigma against women who need concurrent addiction and perinatal care • Insurance billing as perpetuating bias • High cost as perpetuating bias • Long wait list as perpetuating bias • Structural stigma treats drug use as an exceptional condition	[57]
		Discrimination Based on Socioeconomic Status	• Bias against low socioeconomic status women	[52]
		Interpersonal Racism/Bias	• Implicit bias influences providers’ assumptions about what patients might want and direction of care • Bias in provider–patient verbal and non-verbal interactions • Interpersonal racism impeded treatment options and plans • Demeaning, racialized comments • “Othering” language • Language that invokes stereotypes • Racial dimensions of physical and verbal abuse • Racial minority providers report observing more racially demeaning comments and mistreatments • Minority women’s cultural differences viewed as barriers to quality care	[21, 47, 49, 52, 54, 55, 57]
		Institutional Racism	• Belief that institutional racism and implicit bias impact care quality • Providers struggle identifying racism, in their own practice Perception that it is challenging to detect instances of racism and hold colleagues responsible for such behavior • Punitive care and treatment such as unwarranted urine toxicology screening or child protective services involvement for racial minority women • Biased language invokes stereotypes	[21, 53, 54]
		Structural Racism	• Heightened awareness of structural racism • Impact on health outcomes, medical education, and clinician interactions • Stigma is a significant problem across healthcare settings • Need to dismantle racist foundations obstetrics and gynecology	[21, 52, 57]
*MACRO-LEVEL, EXTERNAL CONSTRAINTS AND FACILITATORS* • Transportation, insurance, immigration status, mandatory reporting requirements, and beneficial external collaborations	***Other Systems-Level Factors***	Broken Healthcare System	• Mandatory reporting and CPC involvement affects treating pregnant adolescents • Positive impact of cross-facility collaborations • Positive impact of positive media coverage for adopting innovative patient care systems • Lack of health insurance • Immigration concerns • Positive impact of cross-facility collaborations • Positive impact of positive media coverage for adopting innovative patient care systems • Mandatory reporting laws impact trust and care delivery • Relationship-building and mentorship between implementation teams across hospitals seen as important for scaling shared decision-making intervention.	[49, 55]
		Transportation		[49, 54]
		Insurance & Immigration Status		[49, 51]
		Mandatory Reporting		[51]
		External Partnerships		[56]

#### Being free from harm and mistreatment

Per the key findings from the studies in this review, perinatal healthcare providers describe situations that deprive birthing people of their right to be free from harm and ill-treatment. Providers reported experiencing pregnant and birthing women being physically and verbally abused during facility-based perinatal care [46, 47, 49, 54, 55]. Providers described the nature of physical abuse in the form of grabbing laboring women’s legs with force [46, 54, 58].

Second, verbal abuse during facility-based perinatal care was experienced or reported across four central cadres of healthcare professional roles: [1] nurses [2], midwives [3], doctors, and [4] doulas. Healthcare providers specifically reported acts of verbal abuse, describing experiences such as threats to withhold care from women, passing accusatory comments, blaming women for deficiencies in perinatal care, scolding, screaming, or yelling at and threatening to withhold care from birthing women [46, 54], doing procedures against women’s wishes, and threats to babies’ lives— baby will die [46, 47, 58], and using harsh or rude language, including calling women “stoic” [49, 55].

#### Rapport between providers and women

Healthcare providers commonly describe communication issues with pregnant and birthing women as they navigate perinatal care [46, 49, 50, 55, 58]. Providers narrated an array of experiences, including having skeptical views about cultural competency [50], negative attitudes toward patients having many family members, use of condescending language to describe family or community support practices [49], not introducing themselves to patients [54, 58], lack of trust [55], and the need for providing patient clear explanations [46, 51, 58].

##### Culturally-Sensitive Care

While some healthcare providers held skeptical views about the importance of cultural competency and sensitivity for the quality of prenatal care they can offer women [50], others agreed that providing culturally congruent care improves perinatal outcomes [55]. However, some providers reported a lack of cultural sensitivity and competency in providing maternity care to women [46, 49, 55]. Healthcare providers also described a lack of understanding of the collectivist, supportive nature of women and their community and often use condescending language to describe family or community support practices [49].

##### **Engaging with Effective Communication**

Providers described having experienced situations where colleagues did not introduce themselves to patients [54, 58]. Others also reported their personal biases and opinions as a communication constraint, which makes the patient, especially young women, uncomfortable speaking directly with their doctors [51]. Additionally, some provider statements show ineffective communication, which includes the dismissal of birthing people’s concerns and poor maternity care staff attitudes that show a lack of supportive care that violates women’s autonomy [46, 54, 58]; lack of trust [55]; and providers inability to interpret their patients’ affects [49].

##### **Respecting Women’s Choices and Capabilities to Give Birth**

Providers reported experiencing power dynamics between providers and pregnant patients [51, 53, 56, 58]. Some providers described instances where pregnant women felt that their opinions were not considered by the medical personnel [51], while others reported how physicians sometimes do not listen to birthing people because they do not adhere to the provider’s plan [55]. To address this issue, some healthcare providers suggest encouraging active participation and shared decision-making between providers and patients [46, 56, 58]. Providers also agree that having doulas can empower women to self-advocate and reshape the power dynamics in the patient-provider relationship [53]. However, mandated reporting laws can impact trust and hinder patient-provider communication [51]. Providers often feel conflicted between prioritizing their patients’ needs and following clinical policies and practices that may not always put patients at the center, thus making it challenging to prioritize women’s needs and preferences [52, 58].

#### Meeting professional standards of care

Healthcare providers reported experiences, particularly regarding the prospective provision of information and seeking informed consent [46, 47, 54, 58], and the provision of efficient and effective professional care [50, 51, 54]. Providers described having experienced situations where colleagues performed procedures against patients’ wishes and/or did not give the patient enough time to decide or weigh their options [46, 47, 54]. Also, providers described quality maternity care in terms of completing and communicating the importance of standard required tests [50]. However, some providers’ descriptive narratives of birth mirrored patient descriptions and experiences of injustice, using birth trauma as a euphemism to describe mistreatment [54] and highly valued care characteristics that are indicative of a patient-centered approach [50]. In addition, providers reported their admiration for patients they perceived to have had a higher pain tolerance than normal, relieving them of the responsibility of offering or administering pain relief [49].

#### Providing equitable maternity care

Providers observed instances of stigma and discrimination during the provision of facility-based maternity care based on age [51], race or ethnicity [21, 47–49, 52, 54, 55, 57, 58], socioeconomic status [52], and medical conditions, specifically, addiction [57]. They also acknowledged their negative opinions about adolescent patients’ life choices and pregnancy and found it challenging to provide care when they strongly disagreed with their life decisions [51].

Concerning racism, providers narrated that patients were stigmatized and discriminated against based on their race. While providers viewed demanding White women as needy, demanding Black women were labeled as having a horrible attitude [54]. Also, providers broadly acknowledged that racism influences providers’ perceptions of Black women and their ability to acknowledge and engage Black women as agents of their own bodies and the care provided by providers in hospital settings [21]. Some providers also expressly referred to struggles with their own biases, which implicitly keep them from closely addressing the needs of Black women [55, 57] and biased assumptions about who should or should not have more children [52].

Despite identifying racism as a concern, providers reported struggling to identify how racism impacted their own care practices [21] and that it was challenging to detect instances of racism and bias and hold colleagues responsible for such behavior [53]. Providers also described how hospital visitor policy is not always culturally acceptable to Black families that tend to want more of their family members present [54]. Also, providers recognize structural inequities in the provision of perinatal care [52] and express a heightened awareness of the impact of structural racism on Black women’s health outcomes, medical education, and providers’ interactions with patients [21].

#### Health facility and health system barriers and facilitators

While perinatal healthcare providers discussed individual-level experiences and factors contributing to birthing women’s facility-based experiences, they also discussed health system factors that constrain or enhance care, environment, and culture within a facility [50, 51, 53, 55–57]. However, providers expressed concern about patient caseload, which restricts the time they can spend with each patient [51]. In addition, providers reported that nursing ratios, staffing shortages, and other structural characteristics of healthcare facilities hinder the provision of respectful care [56, 57].

Another health facility and health system factor identified in the reviewed studies is continuity of care and professional labor support. Providers describe their inability to simultaneously manage clinical tasks and provide continuous labor support due to the demands of the clinical roles, such as managing various clinical tasks among multiple patients [53]. Hence, providers acknowledge that the continuous presence of doula staff during delivery makes their work easier as the doulas offer birthing women labor support, which providers are unable to provide effectively [53].

Providers expressed the view that their professional training does not adequately equip them with the necessary skills to manage patients who do not adhere to care plans [55]. Also, some providers reported relying on heuristics from their training and past clinical experiences to address patient needs. On the other hand, providers identified that doula support and advocacy positively impacted healthcare providers’ experience of providing pregnancy-related care and perception of patients’ care experience, allowing medical professionals to focus on clinical tasks [53].

Providers also reported that leadership commitment, positive team culture, and access to multidisciplinary teams are important aspects of prenatal care quality, which enhances providers’ uptake of respectful maternity care interventions [50, 56]. Hospital-level interventions such as regular and transparent feedback from key stakeholders, perceived ease of integrating evidence-based interventions into existing workflows, and programs that challenge providers’ norms and values are factors that enhance shared decision-making around pregnant people’s decisional autonomy [56].

#### Macro-Level, external barriers and facilitators

Providers also reported other external factors that influence the provision of respectful care [49, 51, 54–56]. Providers reported that structural barriers lead to delays in prenatal care initiation and limit women’s reproductive options [49]. Others also referred to the healthcare system as broken, making it difficult for patients to trust providers [55]. Also, providers expressed concerns about the negative impact of mandated reporting laws. They reported that these laws hinder patient-provider communication [51].

Providers identified transportation difficulties as a barrier to preventing patients from attending appointments; however, when minority and low-income patients miss scheduled appointments, they are subjected to drug screens when all they need is transportation [54]. Additionally, some providers reported that insurance and immigration status limited interactions with patients [51]. On the other hand, providers recognized that external partnerships such as relationship-building and mentorship between implementation teams across hospitals are essential for scaling maternity care innovations [56].

### Risk of bias within studies

Quality assessment was completed at the study level using the MMAT (Hong et al., 2018). See Table 4 for a summary of critical appraisal results. On average, each study met al.l five appraisal criteria, with a median of five criteria achieved. All of the studies included in this review achieved a score of 100% (5/5) of the quality appraisal criteria, indicating that the quality of evidence among and within studies is high [21, 47, 49–57].

**Table 4:** Critical appraisal of included studies

First Author (Year)	Screening Questions		Qualitative					Quantitative Randomized Controlled Trial					Quantitative Non-Randomized					Quantitative Descriptive					Mixed Methods					Score
	S1	S2	1.1	1.2	1.3	1.4	1.5	2.1	2.2	2.3	2.4	2.5	3.1	3.2	3.3	3.4	3.5	4.1	4.2	4.3	4.4	4.5	5.1	5.2	5.3	5.4	5.5	
Qualitative																												
Chambers et al. (2022) [21]	Yes	Yes	Yes	Yes	Yes	Yes	Yes	N/A	N/A	N/A	N/A	N/A	N/A	N/A	N/A	N/A	N/A	N/A	N/A	N/A	N/A	N/A	N/A	N/A	N/A	N/A	N/A	100%
Salter et al. (2023) [54]	Yes	Yes	Yes	Yes	Yes	Yes	Yes	N/A	N/A	N/A	N/A	N/A	N/A	N/A	N/A	N/A	N/A	N/A	N/A	N/A	N/A	N/A	N/A	N/A	N/A	N/A	N/A	100%
Decker et al. (2021) [51]	Yes	Yes	Yes	Yes	Yes	Yes	Yes	N/A	N/A	N/A	N/A	N/A	N/A	N/A	N/A	N/A	N/A	N/A	N/A	N/A	N/A	N/A	N/A	N/A	N/A	N/A	N/A	100%
Smith et al. (2022) [55]	Yes	Yes	Yes	Yes	Yes	Yes	Yes	N/A	N/A	N/A	N/A	N/A	N/A	N/A	N/A	N/A	N/A	N/A	N/A	N/A	N/A	N/A	N/A	N/A	N/A	N/A	N/A	100%
Syvertsen et al. (2021) [57]	Yes	Yes	Yes	Yes	Yes	Yes	Yes	N/A	N/A	N/A	N/A	N/A	N/A	N/A	N/A	N/A	N/A	N/A	N/A	N/A	N/A	N/A	N/A	N/A	N/A	N/A	N/A	100%
Reed et al. (2023) [53]	Yes	Yes	Yes	Yes	Yes	Yes	Yes	N/A	N/A	N/A	N/A	N/A	N/A	N/A	N/A	N/A	N/A	N/A	N/A	N/A	N/A	N/A	N/A	N/A	N/A	N/A	N/A	100%
Coley et al. (2018) [50]	Yes	Yes	Yes	Yes	Yes	Yes	Yes	N/A	N/A	N/A	N/A	N/A	N/A	N/A	N/A	N/A	N/A	N/A	N/A	N/A	N/A	N/A	N/A	N/A	N/A	N/A	N/A	100%
Moniz et al. (2022) [52]	Yes	Yes	Yes	Yes	Yes	Yes	Yes	N/A	N/A	N/A	N/A	N/A	N/A	N/A	N/A	N/A	N/A	N/A	N/A	N/A	N/A	N/A	N/A	N/A	N/A	N/A	N/A	100%
Spigel et al. (2022) [56]	Yes	Yes	Yes	Yes	Yes	Yes	Yes	N/A	N/A	N/A	N/A	N/A	N/A	N/A	N/A	N/A	N/A	N/A	N/A	N/A	N/A	N/A	N/A	N/A	N/A	N/A	N/A	100%
Ayers et al. (2018) [49]	Yes	Yes	Yes	Yes	Yes	Yes	Yes	N/A	N/A	N/A	N/A	N/A	N/A	N/A	N/A	N/A	N/A	N/A	N/A	N/A	N/A	N/A	N/A	N/A	N/A	N/A	N/A	100%
Snyder, (2024) [58]	Yes	Yes	Yes	Yes	Yes	Yes	Yes	N/A	N/A	N/A	N/A	N/A	N/A	N/A	N/A	N/A	N/A	N/A	N/A	N/A	N/A	N/A	N/A	N/A	N/A	N/A	N/A	100%
Average Score																												100%
Quantitative Randomized Controlled Trials																												
*None Included*	N/A	N/A	N/A	N/A	N/A	N/A	N/A	N/A	N/A	N/A	N/A	N/A	N/A	N/A	N/A	N/A	N/A	N/A	N/A	N/A	N/A	N/A	N/A	N/A	N/A	N/A	N/A	
Quantitative Non-Randomized																												
Morton et al. (2018) [47]	Yes	Yes	N/A	N/A	N/A	N/A	N/A	N/A	N/A	N/A	N/A	N/A	Yes	Yes	Yes	Yes	Yes	N/A	N/A	N/A	N/A	N/A	N/A	N/A	N/A	N/A	N/A	100%
Hill et al. (2024) [46]	Yes	Yes											Yes	Yes	Yes	Yes	Yes											100%
Quantitative Descriptive																												
*None Included*	N/A	N/A	N/A	N/A	N/A	N/A	N/A	N/A	N/A	N/A	N/A	N/A	N/A	N/A	N/A	N/A	N/A	N/A	N/A	N/A	N/A	N/A	N/A	N/A	N/A	N/A	N/A	
Mixed Method																												
Fachon et al. (2024) [48]	Yes	Yes	N/A	N/A	N/A	N/A	N/A	N/A	N/A	N/A	N/A	N/A	N/A	N/A	N/A	N/A	N/A	N/A	N/A	N/A	N/A	N/A	Yes	Yes	Yes	Yes	Yes	100%
Overall Score																												
Average																												100%

## Discussion

This scoping review identified 14 primary studies published between 2013 and 2024 that examined perinatal healthcare providers’ perspectives on respectful care in the US, revealing paucity of evidence. The studies included a range of providers, such as doulas, physicians, nurses, and certified nurse-midwives, with the majority having sample sizes below 50. Notably, while data from the larger quantitative studies may offer broader generalizability, the smaller qualitative studies provide deeper informational insights.

The findings from this review showed that providers wish to render perinatal care that is respectful and patient-centered. However, their descriptive narratives about physical and verbal abuse, threats to withhold treatment, unconsented perinatal procedures, lack of information about treatment options, and discriminatory interactions mirror the body of evidence on individual pregnant women’s account of mistreatment while navigating perinatal care [60, 61]. In addition, an array of complex structural factors, such as healthcare system culture, facility structural characteristics such as nursing ratios and staffing shortages, provider training, policies for visitors, and mandated drug testing at the hospital level, influence these experiences [51, 55–57]. Thus, these experiences are not merely a function of provider-patient interactions but also a result of non-human, organization-level factors that constrain or enhance the overall perinatal experience. The finding is consistent with Freedman’s view on perinatal care experience as the sum of pregnant women’s interaction with healthcare providers *and* healthcare system failures that sustain those individual-level (in)actions [29, 62]. Beyond these two aspects, however, the review also indicates the influence of macro-level, structural factors outside healthcare facilities (e.g., transportation access, mandatory reporting laws, insurance and immigration issues, and beneficial external partnerships) on providers’ perspectives and experiences of (dis)respectful maternity care.

Overall, providers’ perspectives in this review indicates their awareness of the role of racism and other forms of socioeconomic marginalization as fundamental constraints to the nature of care providers offer Black women and other marginalized groups during the perinatal periods. For example, at the macro level, racism plays a significant role in the uneven distribution of healthcare services and infrastructure, resulting in inadequate healthcare access for Black and other marginalized communities [63]. Thus, minoritized and low-income patients who miss scheduled appointments do not need drug screenings at health centers, as some of the studies in this review found [21, 54]. Instead, they may need transportation—the lack of which underlies the difficulties that prevent patients from attending appointments.

At the institutional or facility level, racism influences facility culture and policies such as visitor policies, which are not always be culturally acceptable to Black families and other patients of color who tend to want more family members present [54], and providers struggle to identify how racism impacted their own care practices [21, 53]. Also, institutional racism is seen in hospital policies resulting in punitive care and treatment, such as unwarranted urine toxicology screening that singles out patients of color if they miss multiple prenatal visits [21, 54]. In addition, interpersonal racism, bias, and discrimination during provider-patient interactions result in substandard care provision. For instance, mistreatment such as verbal abuse and the use of othering language and stereotypes reported by providers in the included studies are racialized [47, 49, 54]. More than one-fifth of providers narrated that patients were often stigmatized and discriminated against based on their race [54]. Considering providers’ awareness of implicit bias in this review, it is primarily White nurses who indicated that they did not believe implicit bias or institutional racism impacts pregnancy-related care [53]. Conversely, doulas and midwives who belong to racial minority groups had 3.3 times higher odds of reporting that they occasionally or often heard care providers make racially demeaning comments and 2.4 times higher odds of witnessing care providers perform extra procedures based on a woman’s race or ethnicity compared to White, non-Hispanic providers [47].

At the provider level, implicit biases and racialized assumptions about Black suffering (e.g., that Black women feel less pain or can withstand pain because of the hardships they have endured in the past) are examples of how perinatal care providers express discrimination and biases during facility-based maternity visits and procedures [21, 64–66]. Providers’ intentional or unintentional mistreatment [28, 29] of pregnant and birthing women during the perinatal period constitutes a violation of their human rights and signifies poor-quality care [30]. However, although women have a fundamental right to high-quality care, the opposite, inequitable care, is prevailing in many facility-based maternity care settings, impacting adverse perinatal outcomes for Black women [24–27]. Quality care rendered by providers entails human and physical resources, referral services, information systems, appropriate technologies, internationally recognized best practices, and emergency service management [34]. Thus, as Hulton et al. [34] observes, while understanding women’s actual experience of maternity care is significant, providers’ perspectives on the service they deliver in maternal health facilities are fundamental to disrupting disrespectful practices and systems, thus ensuring effective care. Generally, this review, indicates that providers are aware of how racism impacts care provision and respectful care [21, 47–49]; however, there is implicit or even explicit avoidance of taking accountability for their own role in perpetuating racism. The disrespect of pregnant women not only impacts the quality of healthcare services provided but also indirectly contributes to maternal mortality and morbidity [24, 67, 68].

### Evidence gaps and recommendations

As discussed earlier, there is a lack of empirical evidence regarding the perspective of perinatal healthcare providers on respectful care during facility-based perinatal care in the U.S. All the publications identified in this area were published in the last six years (2018–2024). To further advance this research area, additional studies employing rigorous research methods are needed to explore the concept of respectful care from the perspective of healthcare providers and to understand how they integrate respectful and dignified care elements in their work to continue developing this area of research.

In this review, of the eleven qualitative studies reviewed, eight did not follow a recognized qualitative methodology. They all appeared to be qualitative descriptions with thematic analysis, except for two studies that used a ground theory approach for data analysis. While qualitative description can be a good starting point for new research areas, it lacks the epistemological foundation that is a hallmark of high-quality qualitative inquiry [69]. Therefore, more empirical research is needed in general, but there is also a need for high-quality, theory-based qualitative research in this emerging area.

Although Black women bear the highest burden of the US maternal mortality and morbidity rate, only 27% of the studies in this review specifically explored providers’ perspectives and experiences regarding the provision of facility-based care to Black women during the perinatal period [8, 9]. Hence, subsequent research could benefit from purposefully sampling providers who serve Black pregnant and birthing people to explore their perspectives. In addition to Black communities bearing the highest maternal mortality burden, other racially minoritized groups, including Indigenous, Native Hawaiian, and Pacific Islander communities, also have experienced high levels of disparities in maternal mortality rates. Thus, additional reviews on respectful maternity care specific to those populations are also warranted.

Also, providers are aware of how racism impacts the provision of respectful care; however, there is implicit or even explicit avoidance of taking accountability for their own role in perpetuating racism. As the evidence from this review indicates, none of the fourteen studies focused on providers admitting to their own practices that are or could be deemed disrespectful to pregnant people, particularly Black individuals. Beyond providers reporting that they witnessed mistreatment of (Black) women by other providers, future studies would benefit from looking at perinatal care providers willing to admit to and share their disrespectful practices. Such research is necessary to fully understand and disrupt dehumanizing maternity care practices and policies.

Finally, contrary to the call to make social science research more theory-informed or theory-oriented [70], half (50%) of the studies did not utilize or explicitly mention using a theoretical or conceptual framework. Of the half 50% who used a theoretical or conceptual framework, only 18% of them employed a framework that explicitly offered a concrete vision of what a dignified maternity care experience would entail or look like. Therefore, this review’s findings about the limited practice of theory-oriented research suggest an opportunity to engage in research that is theory-informed or oriented toward theory-building.

### Recommendation for practice

Hospitals could use this review’s findings to create realistic scenarios in perinatal care settings. These scenarios can help guide providers through activities that foster critical thinking and anti-racist approaches to patient care. These providers’ experience support the need for racial equity training to address not only implicit biases but also the history of institutional racism and multilevel tools that clinicians and healthcare institutions can use to improve the care experiences and outcomes of Black and other minoritized women.

In addition, providers should consider ways engaging with Black and other minoritized communities to better understand their culture and to foster more trusting communication with these communities. For example, healthcare providers could engage an advisory board of minoritized community members to help advise them on care, program design, and implementation. Further, as proposed in the Association of Women’s Health, Obstetric, and Neonatal Nursing [71] position statement on respectful maternity care, hospitals can adopt and utilize the respectful maternity care framework for evidence-based guidelines for clinical practice.

## Limitations

This scoping review has several limitations. The potential risk of publication bias limits the findings of this scoping review, as it did not include unpublished works such as articles, dissertations, and conference presentations. Second, given that home births are conceptually different from facility-based birth experiences [24], the review did not include studies exploring provider perspectives of maternity care experience during home birth. Additionally, as was explained in the introduction, this scoping review focused on facility-based versus home-based perinatal care because a vast majority of perinatal care in the US occurs in hospitals. Due to the multidisciplinary nature and scope of the phenomenon of interest in this review, the search might have missed some relevant articles. The review was also limited to studies conducted in the US and those published in English between 2013 and 2024.

## Conclusion

Racial disparities exist in the maternal mortality and morbidity crisis in the US, and Black women bear the highest burden of this preventable challenge [8, 9]. The World Health Organization observes that skilled facility-based maternity care throughout pregnancy, labor, and postpartum is essential for addressing this challenge [16, 68]. This scoping review finds that providers’ maternity care provision experience is generally negative, manifesting as mistreatment of pregnant people. Providers’ recounted experiences of mistreatment received by pregnant women during pregnancy and childbirth are not only shaped by the quality of provider-patient interactions and procedures but also by healthcare facility-level conditions that constrain or sustain those interactions. Beyond this, the review finds that societal-level inequities (e.g., immigration, insurance, and transportation) impose additional burdens on providers’ ability to offer dignified maternity care. In all, a common thread that runs through the different layers of mistreatment of pregnant women reported by providers is the pervasiveness of racism and other forms of social marginalization permeating all levels of facility-based maternity care in the US. Interventions that address these challenges would need to address not just the biased interactions between providers and pregnant women but also the structural inequalities that conspire against both providers and patients— especially Black birthing people.

## Supplementary Information

Supplementary Material 1.

## Data Availability

No datasets were generated or analysed during the current study.
