# Supplementary material for: An exploration of perinatal healthcare providers’ perspectives on respectful maternity care in the United States: a scoping review
Source: BMC Pregnancy Childbirth. 2025 Nov 4;25:1165. doi: 10.1186/s12884-025-08247-y (PMC12584471; doi:10.1186/s12884-025-08247-y)

**SUPPLEMENTAL MATERIALS**

**Title:** An Exploration of Perinatal Healthcare Providers’ Perspectives on Respectful Maternity Care in the United States: A Scoping Review

**Authors:**

Celestine Yayra Ofori-Parku BSc, Dip.M, RM (**Corresponding Author**)

PhD Candidate

Department of Family Health Care Nursing

School of Nursing, University of California San Francisco,

490 Illinois Street

San Francisco, CA 94158

United States

[Celestine.Ofori-Parku@ucsf.edu](mailto:Celestine.Ofori-Parku@ucsf.edu)

Orlando Omar Harris PhD, RN, FNP, MPH, FAAN

Community Health Systems,

School of Nursing, University of California San Francisco

490 Illinois Street, #92S

San Francisco, CA 94158

United States

Kimberly Baltzell RN, PhD, MS

Department of Family Health Care Nursing

School of Nursing, University of California San Francisco,

550 16th Street, #3507
San Francisco, CA 94158

United States

Emily Little PhD

Nurturely

56 East 15th Ave

Eugene, Oregon 97401

United States

Ifeyinwa V. Asiodu PhD, RN, IBCLC, FAAN

Department of Family Health Care Nursing

School of Nursing, University of California San Francisco,

490 Illinois Street, #92J,

San Francisco, CA 94158

United States

## Appendices

### Appendix I: Preferred Reporting Items for Systematic reviews and Meta-Analyses Extension for Scoping Reviews (PRISMA-ScR) Checklist

| **SECTION** | **ITEM** | **PRISMA-ScR CHECKLIST ITEM** | **REPORTED ON PAGE #** |
| --- | --- | --- | --- |
| **TITLE** | | | |
| Title | 1 | Identify the report as a scoping review. | 1 |
| **ABSTRACT** | | | |
| Structured summary | 2 | Provide a structured summary that includes (as applicable): background, objectives, eligibility criteria, sources of evidence, charting methods, results, and conclusions that relate to the review questions and objectives. | 2 |
| **INTRODUCTION** | | | |
| Rationale | 3 | Describe the rationale for the review in the context of what is already known. Explain why the review questions/objectives lend themselves to a scoping review approach. | 3 |
| Objectives | 4 | Provide an explicit statement of the questions and objectives being addressed with reference to their key elements (e.g., population or participants, concepts, and context) or other relevant key elements used to conceptualize the review questions and/or objectives. | 5 |
| **METHODS** | | | |
| Protocol and registration | 5 | Indicate whether a review protocol exists; state if and where it can be accessed (e.g., a Web address); and if available, provide registration information, including the registration number. | 7 |
| Eligibility criteria | 6 | Specify characteristics of the sources of evidence used as eligibility criteria (e.g., years considered, language, and publication status), and provide a rationale. | 7 |
| Information sources* | 7 | Describe all information sources in the search (e.g., databases with dates of coverage and contact with authors to identify additional sources), as well as the date the most recent search was executed. | 8 |
| Search | 8 | Present the full electronic search strategy for at least 1 database, including any limits used, such that it could be repeated. | 8 |
| Selection of sources of evidence† | 9 | State the process for selecting sources of evidence (i.e., screening and eligibility) included in the scoping review. | 8 |
| Data charting process‡ | 10 | Describe the methods of charting data from the included sources of evidence (e.g., calibrated forms or forms that have been tested by the team before their use, and whether data charting was done independently or in duplicate) and any processes for obtaining and confirming data from investigators. | 9 |
| Data items | 11 | List and define all variables for which data were sought and any assumptions and simplifications made. | 9 |
| Critical appraisal of individual sources of evidence§ | 12 | If done, provide a rationale for conducting a critical appraisal of included sources of evidence; describe the methods used and how this information was used in any data synthesis (if appropriate). | 10 |
| Synthesis of results | 13 | Describe the methods of handling and summarizing the data that were charted. | 9 |
| **RESULTS** | | | |
| Selection of sources of evidence | 14 | Give numbers of sources of evidence screened, assessed for eligibility, and included in the review, with reasons for exclusions at each stage, ideally using a flow diagram. | 11 |
| Characteristics of sources of evidence | 15 | For each source of evidence, present characteristics for which data were charted and provide the citations. | 13 |
| Critical appraisal within sources of evidence | 16 | If done, present data on critical appraisal of included sources of evidence (see item 12). | 24 |
| Results of individual sources of evidence | 17 | For each included source of evidence, present the relevant data that were charted that relate to the review questions and objectives. | 13 |
| Synthesis of results | 18 | Summarize and/or present the charting results as they relate to the review questions and objectives. | 16-23 |
| **DISCUSSION** | | | |
| Summary of evidence | 19 | Summarize the main results (including an overview of concepts, themes, and types of evidence available), link to the review questions and objectives, and consider the relevance to key groups. | 24 |
| Limitations | 20 | Discuss the limitations of the scoping review process. | 29 |
| Conclusions | 21 | Provide a general interpretation of the results with respect to the review questions and objectives, as well as potential implications and/or next steps. | 30 |
| **FUNDING** | | | |
| Funding | 22 | Describe sources of funding for the included sources of evidence, as well as sources of funding for the scoping review. Describe the role of the funders of the scoping review. | 31 |

*From:* Tricco AC, Lillie E, Zarin W, O'Brien KK, Colquhoun H, Levac D, et al. PRISMA Extension for Scoping Reviews (PRISMAScR): Checklist and Explanation. Ann Intern Med. 2018;169:467–473. [(56,57)](http://annals.org/aim/fullarticle/2700389/prisma-extension-scoping-reviews-prisma-scr-checklist-explanation).

**Appendix II: Eligibility Criteria**

|  | Inclusion criteria | Exclusion criteria |
| --- | --- | --- |
| Participants | Perinatal healthcare providers: Physicians, nurses, APRNs, and midwives in the United States who work in a facility-based setting. | - Non-perinatal healthcare providers, perinatal healthcare providers, and midwives outside the US - Perinatal healthcare providers in non-facility-based settings. |
| Concepts | - Provider perspectives and experiences of disrespect & abuse and obstetric violence in pregnancy and birthing - Provider perspectives and experiences of respectful maternity care (RMC). - Perspectives on the barriers to the provision of RMC - Perspectives on the facilitators to the provision of RMC - How providers incorporate the elements of RMC into their everyday practice - Article characteristics: year of publication, authors, author affiliations - Provider characteristics - Institutional constraints - Facility policies, practices, resources - Structural/systemic racism - Implicit bias training - Measures taken by individual studies to ensure rigor or trustworthiness of the studies | - Perspectives of birthing individuals. - Results that talk about the perspectives and experiences of non-providers - Results about perspectives and experiences providers outside the United States/healthcare settings. |
| Context | - Facility-based maternity care - Setting: United States - Time frame: Studies published between 2013-2024. - Language: Full text in English. - Peer-reviewed journals | Non-facility-based maternity care settings  Home birth |
| Type of Sources of Evidence Design | - Qualitative studies: Critical ethnography, grounded theory, case studies, interpretive phenomenology, comparative qualitative studies. Quantitative studies: Cross-sectional, case control, and intervention studies focusing on providers’ perspectives and experiences of (dis) respectful maternity care. | Editorials, commentary, viewpoints, protocols, practice guides, instrument development for women’s experience, and systematic or other reviews. |

### Appendix III: Search Strategy

**PubMed**

Updated March 5, 2025

| # | SEARCH | NUMBER OF PAPERS |
| --- | --- | --- |
| 1 | (Clinicians OR obstetricians OR healthcare providers OR providers OR doctors OR midwives OR nurses) | 6,069,891 |
| 2 | perspective OR perception OR experience OR knowledge | 3,243,166 |
| 3 | (Respectful maternity care OR Person-centered maternity care OR compassionate maternity care OR Humanization in birth OR mistreatment OR disrespectful Maternity care OR obstetric violence) | 99,452 |
| 4 | # 1AND #2 AND #3 | 13,002 |
| 5 | #4 AND Filters applied: in the past 10 years (2013- 2024) | 9,364 |
| 6 | #5 AND Filters applied: in the United States | 1,704 |
|  | #6 AND  Free full text, Full text, Comparative Study, Controlled Clinical Trial, Evaluation Study, Government Publication, Interview, Observational Study, Randomized Controlled Trial, Validation Study, English, from 2013/1/1 - 2024/12/31 | 108 |

**Embase**

Updated March 5, 2025

| # | SEARCH | NUMBER OF PAPERS |
| --- | --- | --- |
| 1 | (Clinicians OR obstetricians OR healthcare providers OR providers OR doctors OR midwives OR nurses) | 1,345.061 |
| 2 | perspective OR perception OR experience OR knowledge | 3,729,082 |
| 3 | (Respectful maternity care OR Person-centered maternity care OR compassionate maternity care OR Humanization in birth OR mistreatment OR disrespectful Maternity care OR obstetric violence) | 6,178 |
| 4 | #1 AND #2 AND #3 | 2,140 |
| 5 | #4 AND Filters applied: in the past 10 years (2013- 2024) | 1,737 |
| 6 | #5 AND Filters applied: in the United States | 474 |
| 7 | #6 AND ('case study'/de OR 'clinical article'/de OR 'clinical study'/de OR 'cohort analysis'/de OR 'comparative effectiveness'/de OR 'comparative study'/de OR 'controlled study'/de OR 'cross sectional study'/de OR 'delphi study'/de OR 'evidence based practice'/de OR 'exploratory research'/de OR 'feasibility study'/de OR 'grounded theory'/de OR 'human'/de OR 'interview'/de OR 'longitudinal study'/de OR 'major clinical study'/de OR 'observational study'/de OR 'open ended questionnaire'/de OR 'participatory research'/de OR 'pilot study'/de OR 'prospective study'/de OR 'qualitative research'/de OR 'quantitative study'/de OR 'questionnaire'/de OR 'retrospective study'/de OR 'semi structured interview'/de OR 'structured interview'/de OR 'structured questionnaire'/de) | 266 |

**Web of Science**

Updated March 5, 2025

| # | SEARCH | NUMBER OF PAPERS |
| --- | --- | --- |
| 1 | (Clinicians OR obstetricians OR healthcare providers OR providers OR doctors OR midwives OR nurses) | 1,357,654 |
| 2 | perspective OR perception OR experience OR knowledge | 7,459,980 |
| 3 | (Respectful maternity care OR Person-centered maternity care OR compassionate maternity care OR Humanization in birth OR mistreatment OR disrespectful Maternity care OR obstetric violence) | 7,303 |
| 4 | #1 AND #2 AND #3 | 1,334 |
| 5 | #4 AND Filters applied: in the past 10 years (2013- 2024) | 1,169 |
| 6 | #5 AND Filters applied: in the United States | 482 |
| 7 | #6 AND Filters applied: conference proceedings, article, early access, English. | 431 |

**CINAHL**

Updated March 5, 2025

| # | SEARCH | NUMBER OF PAPERS |
| --- | --- | --- |
| 1 | (Clinicians OR obstetricians OR healthcare providers OR providers OR doctors OR midwives OR nurses) | 926,768 |
| 2 | perspective OR perception OR experience OR knowledge | 1,016,865 |
| 3 | (Respectful maternity care OR Person-centered maternity care OR compassionate maternity care OR Humanization in birth OR mistreatment OR disrespectful Maternity care OR obstetric violence) | 1,980 |
| 4 | #1 AND #2 AND #3 | 400 |
| 5 | #4 AND Filters applied: in the past 10 years (2013- 2024) | 363 |
| 6 | #5 AND Filters applied: in the United States | 87 |
| 7 | #6 AND Filters applied: Academic journals and English language | 82 |

### Appendix IV: Mixed Methods Appraisal Tool (MMAT), Version 2018


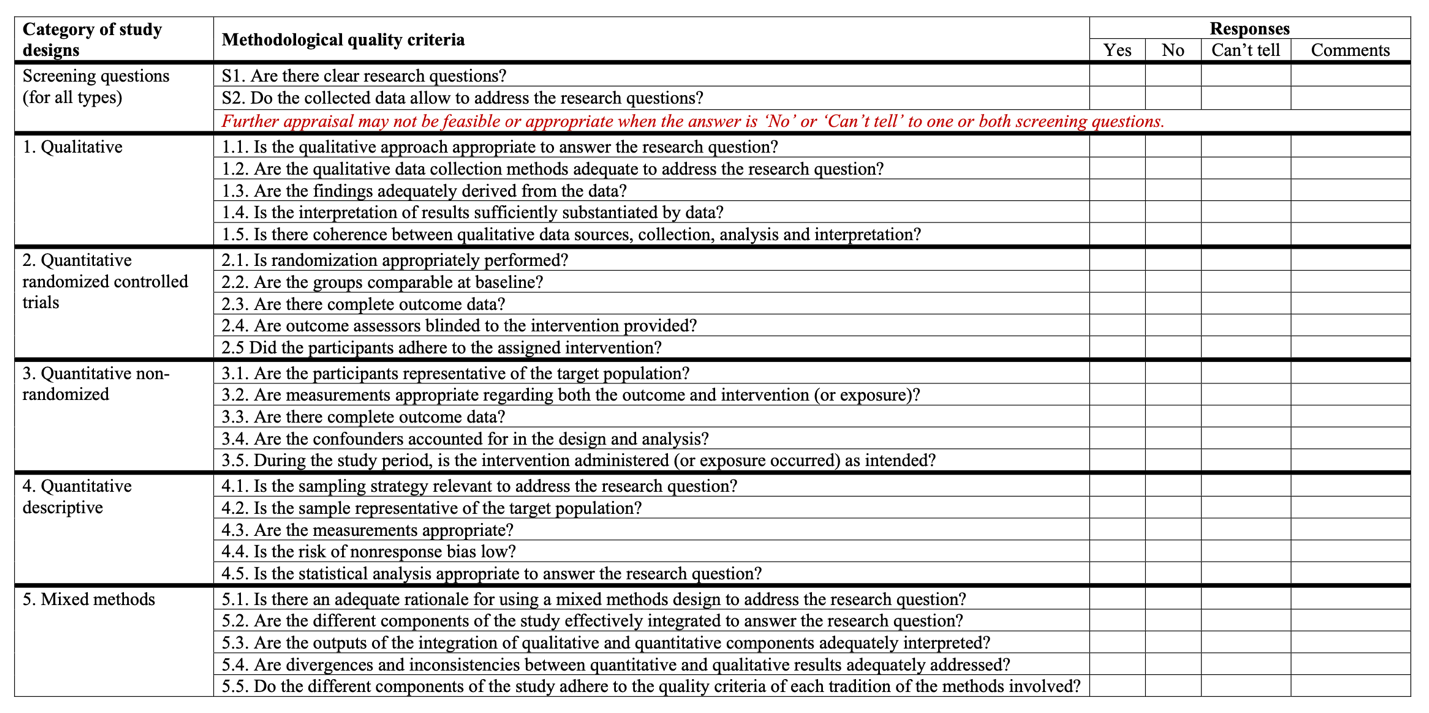

Supplement: Supplementary file 1 — Supplementary Material 1. [file 12884_2025_8247_MOESM1_ESM.docx]
